# Decoy Extracellular Vesicles Overcome Triple‐Negative Breast Cancer Heterogeneity via Membrane‐Cytoplasm‐Mitochondria Cascade Targeting

**DOI:** 10.1002/advs.202507975

**Published:** 2025-08-04

**Authors:** Chuanrong Chen, Ming Shen, Xiaofeng Wan, Lili Sheng, Na Hao, Man Li, Menglin Xu, Yang He, Jiali Zhang

**Affiliations:** ^1^ Department of Oncology Yijishan Hospital of Wannan Medical College Wuhu 240001 China; ^2^ National Health Commission (NHC) Key Laboratory of Reproduction Regulation Shanghai Institute for Biomedical and Pharmaceutical Technologies Shanghai 200032 China; ^3^ State Key Laboratory of Oncogenes and Related Genes Shanghai Cancer Institute Renji Hospital School of Medicine Shanghai Jiao Tong University Shanghai 200032 China

**Keywords:** cascade target, decoy extracellular vesicles, heterogeneity, multi‐immune checkpoint block, triple‐negative breast cancer

## Abstract

Triple‐negative breast cancer (TNBC) exhibits multilevel heterogeneity, including differences in stemness, immune checkpoint (IC) upregulation, and metabolic plasticity, which has resulted in a lack, of comprehensive treatment strategies. Extracellular vesicles (EVs) from exhausted T cells contain high levels of ICs, which allow them to target and block multiple IC ligands in tumor cells, including cancer stem cells (CSCs). TEAD4 is identified as a pivotal gene facilitating CSC growth and immune escape. Consequently, a novel EV delivery system (siT/MOF@EVs), which targets the tumor cell membrane–cytoplasm–mitochondria cascade, is engineered to codeliver TEAD4‐siRNA (siTEAD4) and a mitochondria‐targeting metal–organic framework (T/MOF) to overcome tumor heterogeneity. Extracellularly, EVs act as “decoys” by facilitating the binding of IC ligands to target tumor cells, particularly CSCs, and blocking multiple ICs. Within the cytoplasm, the siTEAD4 adsorbed on the T/MOF surface is released, inhibiting CSCs and overcoming immunotherapy resistance. In mitochondria, cascade catalysis by nanozymes that bind monoatomic Pd and Mn^2+^ triggers an oxidative burst. Additionally, siTEAD4 and T/MOF synergistically block aerobic glycolysis and oxidative phosphorylation and induce immunogenic tumor cell death. siT/MOF@EVs overcome TNBC heterogeneity, inhibit tumor growth and metastasis, and induce protective immune memory in an orthotopic TNBC model without systemic toxicity. Significant therapeutic effects are also observed in a patient‐derived organoid xenograft (PDOX) model.

## Graphical abstract

siT/MOF@EVs overcome TNBC heterogeneity through a tumor cell membrane–cytoplasm–mitochondria cascade effect, triggering a self‐reinforcing anti‐tumor immune cycle and durable immune memory. The system enables sequential targeting of membrane checkpoints ligands (decoy effect), cytoplasmic siRNA delivery (CSC suppression), and mitochondrial MOF accumulation (STING activation). Multimodal action combines immune checkpoint blockade, metabolic disruption, and ROS‐mediated broad‐spectrum cytotoxicity.

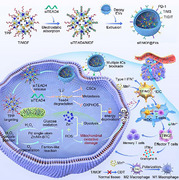

## 1 Background

Triple‐negative breast cancer (TNBC), characterized by early onset, high aggressiveness, and a lack of therapeutic targets, represents the most prognostically unfavorable breast cancer subtype.^[^ ^1^ ^]^

A critical barrier to effective TNBC treatment lies in cancer stem cells (CSCs), a self‐renewing subpopulation that drives tumor heterogeneity and fosters an immunosuppressive tumor microenvironment (ITM) through immune modulation and adaptive plasticity.^[^ ^2^ ^]^ Current therapeutic strategies fail to eradicate both CSCs and non‐CSCs concurrently, underscoring the urgent need for innovative approaches.^[^ ^2^, ^3^ ^]^

Mitochondria, the central hubs for energy metabolism and apoptosis regulation, exhibit structural and functional aberrations in malignant cells, rendering these cells vulnerable to mitochondrial disruption.^[^ ^4^, ^5^ ^]^ Chemodynamic therapy (CDT) leverages tumor‐specific H_2_O_2_ overload to generate cytotoxic reactive oxygen species (ROS) via Fenton or Fenton‐like reactions catalyzed by transition metals.^[^ ^6^ ^]^ Oxidative damage induced by excessive ROS can alter mitochondrial membrane permeability, leading to the release of proapoptotic molecules and mtDNA into the cytoplasm to activate cGAS–STING‐dependent type I interferons, which increases the immunogenicity of tumor cells and endoplasmic reticulum stress to promote immunogenic cell death (ICD) in tumors.^[^ ^7^, ^8^ ^]^

Metal‒organic frameworks (MOFs), engineered from metal ions and organic ligands, offer advantages such as tunable porosity, high drug loading capacity, and biodegradability.^[^ ^9^ ^]^ While MOF nanozymes mimic catalysis by enzymes with superior cost‐effectiveness and stability compared with natural enzymes, their clinical translation is hindered by suboptimal ROS generation efficiency.^[^ ^10^ ^]^ This limitation is exacerbated in CSCs, where elevated aldehyde dehydrogenase (ALDH) activity increases ROS scavenging and aldehyde detoxification.^[^ ^10^, ^11^, ^12^ ^]^ Recent advances in single‐atom nanozymes, particularly palladium‐based catalysts with orders of magnitude increased activity, present a promising strategy to increase MOF therapeutic efficacy.^[^ ^13^ ^]^

The abnormal activation of genes affecting antigen presentation and immune cell activation in tumor cells is an inherent mechanism that leads to immunotherapy resistance. TEAD4, a pivotal transcriptional coactivator, partners with YAP to drive the upregulation of immunosuppressive cytokines (e.g., CCL2 and CSF1), stemness‐associated genes (e.g., SOX2 and OCT4), and IC ligands (e.g., PD‐L1), thereby sustaining CSC stemness and facilitating immune escape.^[^ ^14^, ^15^, ^16^ ^]^ In addition, TEAD4 regulates mitochondrial dynamics and cell metabolism by modulating the expression of genes encoding enzymes in the mitochondrial–nuclear electron transport chain (ETC), linking immune evasion and CSCs to metabolic plasticity.^[^ ^17^, ^18^, ^19^ ^]^ Clinically, TEAD4 is markedly overexpressed in TNBC tissues, underscoring its potential as a dual‐action therapeutic target to suppress stemness and restore immune surveillance while sparing normal cells.

Tumor cells, particularly CSCs, drive immunotherapy resistance by upregulating multiple IC ligands to engage surface receptors (PD‐1, TIM‐3, and TIGIT) on immune cells to induce T‐cell exhaustion, M2 macrophage polarization, and dendritic cell (DC) suppression.^[^ ^20^, ^21^ ^]^ While clinical studies have demonstrated that combined blockade of PD‐1/PD‐L1 with TIM‐3 or TIGIT inhibitors improves tumor remission rates compared with monotherapy, multiantibody regimens often provoke severe systemic toxicity.^[^ ^22^, ^23^ ^]^ T‐cell‐derived extracellular vesicles (EVs) intrinsically exhibit tumor‐targeting properties mediated by cell membrane proteins such as lymphocyte function‐associated antigen 1 (LFA‐1), which facilitates their adhesion to the tumor vasculature.^[^ ^24^, ^25^ ^]^ Our group and others have demonstrated that EVs released by activated T cells not only encapsulate antitumor cytokines and cytotoxic molecules but also display membrane‐anchored PD‐1.^[^ ^26^, ^27^ ^]^ Surface‐expressed PD‐1 competitively binds to tumor‐expressed PD‐L1, thereby blocking PD‐L1‐mediated immunosuppression and enhancing antitumor immunity. Our findings demonstrate that modulating T‐cell activation states to produce functionalized EVs offers a simpler, more effective, and biologically safer alternative to conventional genetic or chemical modification approaches. While exhausted T cells naturally upregulate multiple ICs, their therapeutic application has been limited by their poor expansion capacity. In this study, we developed a method combining continuous CD8^+^ T‐cell activation through anti‐CD3/CD28 antibody (αCD3/αCD28) stimulation with interleukin‐10 (IL‐10) supplementation, which successfully expanded the exhausted T‐cell population. These expanded cells generated EVs with various IC proteins enriched on their surface. Remarkably, these multi‐IC‐bearing EVs function as molecular decoys that specifically target tumor cells, particularly CSCs highly expressing ICs, through competitive binding. This specific blockade strategy effectively inhibits multiple tumor immune evasion pathways simultaneously while maintaining a favorable biosafety profile, presenting a novel solution to overcome cancer cell resistance to immunotherapy.

**Scheme 1:**
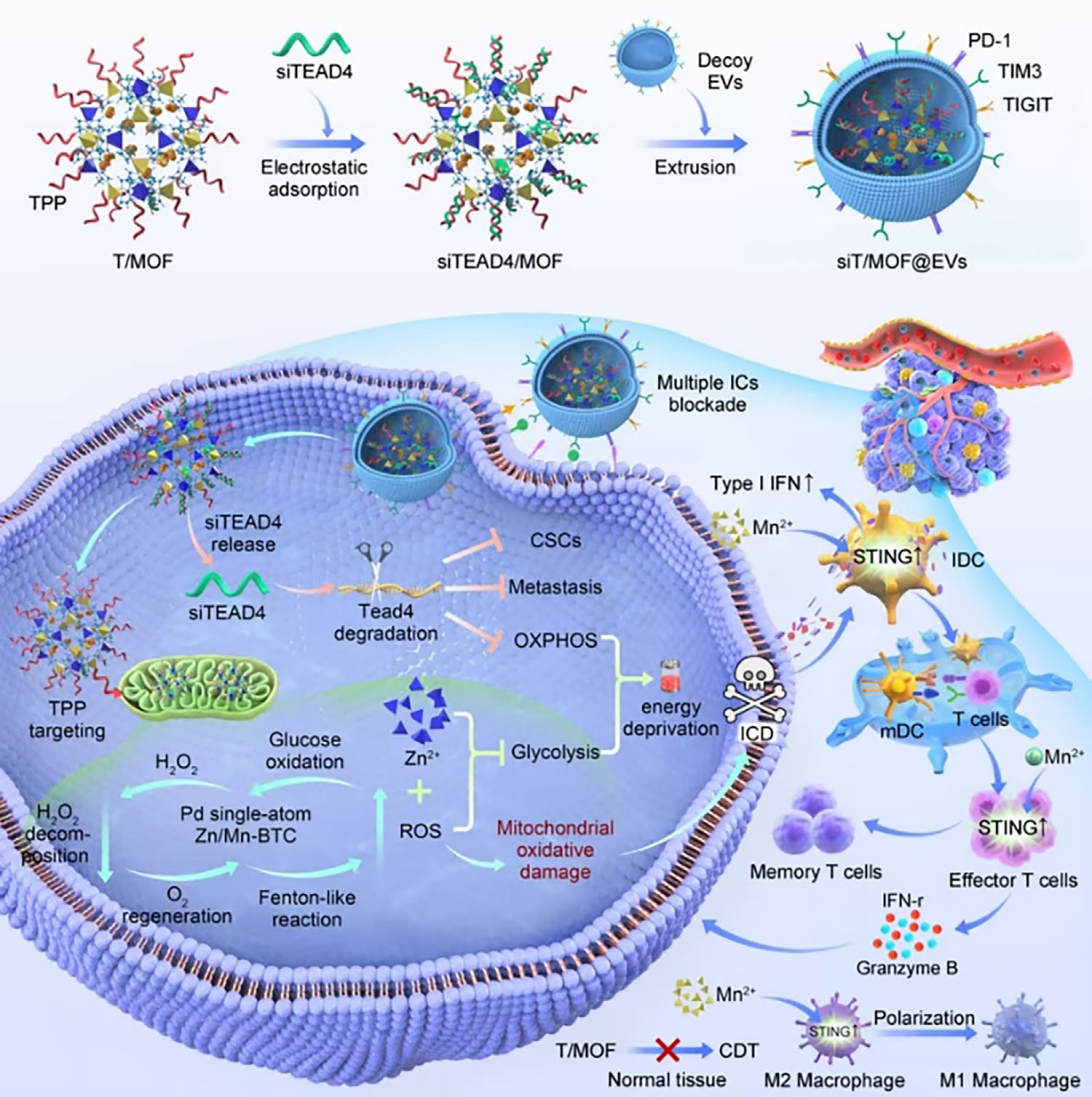
siT/MOF@EVs overcame the heterogeneity of TNBC and elicited a positive feedback antitumor immune cycle and protective immune memory response owing to the antitumor effect of the tumor cell membrane–cytoplasm–mitochondria cascade.

In this study, we prepared a Zn/Mn‐MOF that specifically targets tumor cell mitochondria as the primary carrier, loaded monoatomic Pd‐binding nanozymes into the pores, attached TEAD4‐siRNA (siTEAD4) to the surface via electrostatic adsorption, and then performed decoy EVs encapsulation to construct an EV delivery system that targets the cell membrane‒cytoplasm‒mitochondria cascade. In addition to tumor cells, decoy EVs that highly express ICs target tumor cells and mediate blockade of multiple ICs. siTEAD4 adsorbed on the MOF surface was released inside tumor cells owing to the low pH of the cytoplasm to inhibit tumor metastasis and tumor cell stemness; then, the MOF modified with a tumor mitochondria‐targeting group entered the mitochondria, where monoatomic Pd and Mn^2+^ catalyzed the production of an oxidative burst, which induced mitochondrial oxidative damage. In addition, Zn^2+^ and excess ROS synergistically inhibited tumor glycolysis, whereas siTEAD4 also inhibited oxidative phosphorylation (OXPHOS), overcoming tumor metabolic plasticity. These effects synergized with excess intracellular ROS to trigger ICD and induce a widespread immune response in an orthotopic TNBC mouse model. In addition, siT/MOF@EVs significantly inhibited tumor growth in a humanized patient‐derived organoid xenograft (PDOX) model (Scheme 1).

## 2 Results and Discussion

### 2.1 Extracellular Vesicles from Exhausted CD8^+^ T Cells Exert Decoy Effects to Mediate the Blockade of Multiple ICs

**Figure 1:**
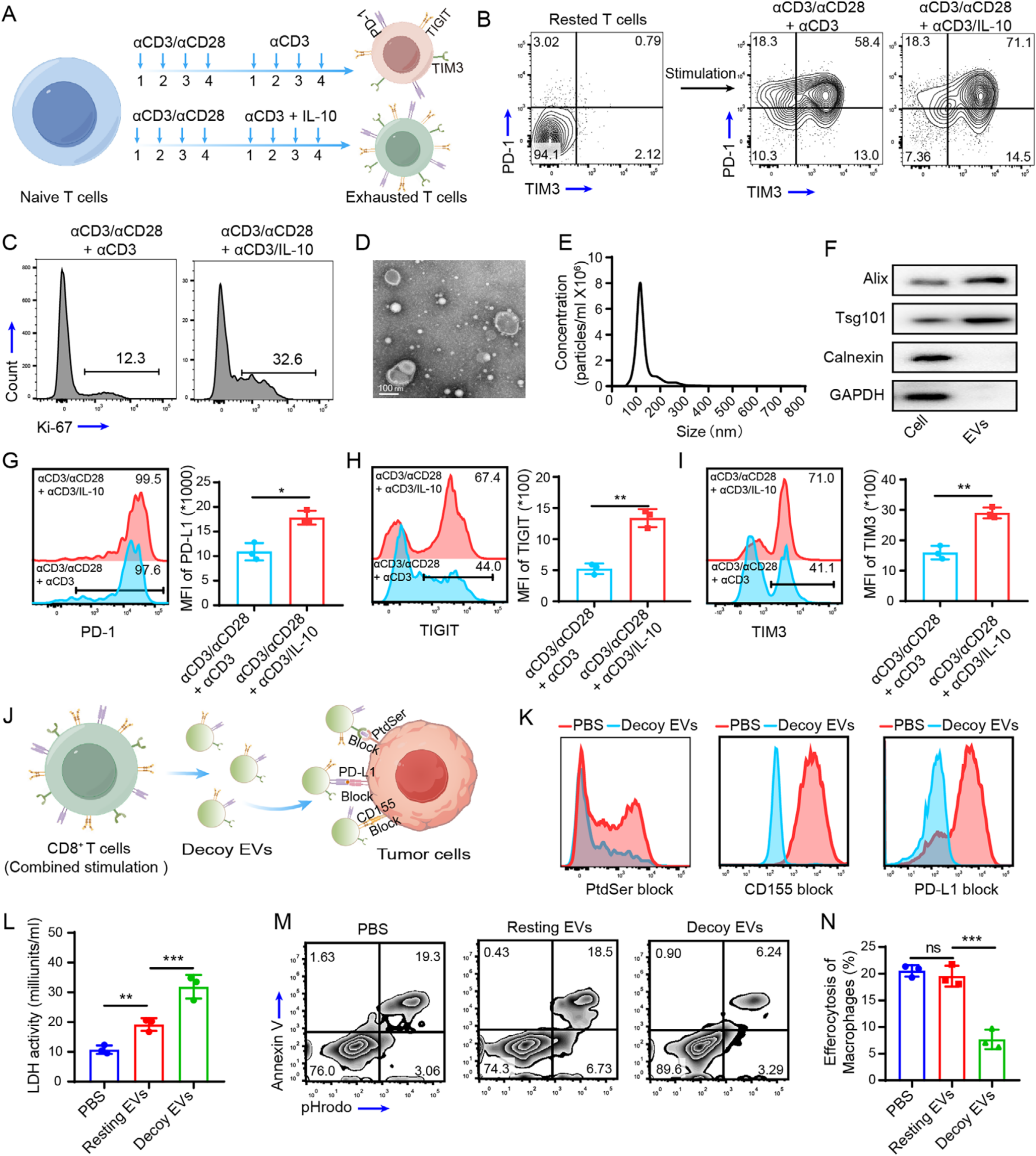
Decoy EVs block the PD‐1/PD‐L1, TIGIT/CD155, and PtdSer/TIM‐3 immune checkpoint axes to modulate the function of CD8^+^ T cells and macrophages. A) Schematic of the induction of CD8^+^ T‐cell exhaustion. Naïve CD8^+^ T cells were stimulated with αCD3/αCD28 for 4 days, followed by continued stimulation with αCD3 or αCD3+IL‐10 for an additional 4 days. B) Flow cytometry analysis of exhausted CD8^+^ T cells (PD‐1^+^TIM‐3^+^). C) Expression of the proliferation marker Ki‐67 in exhausted CD8^+^ T cells after different types of stimulation. D) TEM image of EVs. E) NTA analysis of EVs. (F) Western blot detection of EVs marker proteins. G–I) Flow cytometry analysis of the expression of the key immune checkpoints PD‐1 (G), TIGIT (H), and TIM‐3 (I) in EVs derived from exhausted CD8^+^ T cells. J) Schematic of decoy EVs blocking the surface IC ligands of tumor cells. K) Decoy EVs were incubated with 4T1 cells for 30 min, after which the cells were collected and stained with the corresponding fluorescently labeled antibodies, and flow cytometry analysis of the binding of decoy EVs to antibodies against the immune checkpoint ligands PtdSer, CD155, and PD‐L1 was performed. For PtdSer analysis, 4T1 cells were irradiated with a 254 nm UV lamp for 15 min to induce apoptosis before exposure to PtdSer. L) Coculture of tumor cells with activated OT‐1 CD8^+^ T cells in the presence of 8 µm OVA peptide, PBS, resting EVs, or decoy EVs for 24 h. The cytotoxicity of CD8^+^ T cells was evaluated by measuring the LDH concentration in the supernatant. M) BMDMs were pretreated with resting EVs or decoy EVs for 24 h, followed by coculture with apoptotic 4T1 cells for 4 h. Phagocytosis of apoptotic 4T1 cells by macrophages was analyzed by flow cytometry. N) Quantification of the efferocytosis percentages from panel M. The data are presented as the means ± SD (n = 3). p‐values are calculated using one‐way ANOVA with Tukey's multiple comparison. ^*^ *p* < 0.05, ^**^ *p* < 0.01, ^***^ *p* < 0.001.

Clinical evidence has demonstrated the superior efficacy of combined PD‐1/PD‐L1 and TIM‐3 or TIGIT blockade than single‐IC inhibition.^[^ ^22^, ^23^ ^]^ However, multi‐IC blockade antibody therapies are often accompanied by more severe toxicity. Inspired by the high expression of multiple ICs in exhausted T cells, we hypothesized that EVs derived from exhausted T cells may act as decoys to block multiple ICs.^[^ ^28^, ^29^, ^30^, ^31^ ^]^ However, exhausted T cell expansion is difficult, making it challenging to produce EVs on a large scale. In this study, we induced CD8^+^ T‐cell exhaustion by in vitro stimulation with αCD3/αCD28 plus IL‐10 (**Figure** **1**A). The results revealed that after treatment with IL‐10, the proportion of exhausted CD8^+^ T cells (PD‐1^+^TIM‐3^+^) increased from 58.4% to 71.1% (Figure 1B). Additionally, in the αCD3/αCD28 plus IL‐10 group, PD‐1^+^TIM‐3^+^ T cells also presented increased Ki‐67 expression, indicating enhanced proliferative capacity (Figure 1C). Transmission electron microscopy (TEM) confirmed that the extracted EVs exhibited a typical saucer shape (Figure 1D). Nanoparticle tracking analysis (NTA) revealed an average size of ≈115.4 ± 9.3 nm (Figure 1E). Furthermore, Western blotting revealed the expression of membrane surface protein markers (Alix and Tsg101) in the EVs, whereas cytoplasmic protein markers (GAPDH and Calnexin) were absent (Figure 1F), further confirming the successful extraction of the EVs. Flow cytometry analysis of IC expression on the surface of EVs revealed the high expression of PD‐1, TIM‐3, and TIGIT, with a more significant upregulation upon IL‐10 stimulation (Figure 1G–I). Importantly, pretreatment of 4T1 cells with decoy EVs effectively blocked the binding of tumor cell surface PD‐L1 (PD‐1 ligand), CD155 (TIGIT ligand), and PtdSter (TIM‐3 ligand) with the specific antibodies, indicating the potential of decoy EVs for combined blockade of multiple ICs (Figure 1J,K). CSCs exhibited increased uptake of decoy EVs because of their increased expression of IC ligands, while blocking PD‐1/TIGIT/Tim‐3 on EVs can significantly inhibit EVs uptake by tumor cells (Figure S1, Supporting Information). To analyze the immunotherapeutic effects of decoy EVs, 4T1 cells were cocultured with OT‐1 CD8^+^ T cells in the presence of ovalbumin (OVA) peptide. Compared with those in cells cultured with EVs derived from resting T cells (not activated), LDH levels were significantly greater in the decoy EVs group, suggesting that decoy EVs‐mediated blockade of multiple ICs significantly enhanced the toxic effects of CD8^+^ T cells to tumor cells (Figure 1L). Macrophage efferocytosis removes apoptotic tumor cells, preventing the release of damage‐associated molecular patterns (DAMPs) into the tumor microenvironment (TME) and promoting M2 macrophage polarization, leading to immune‐silent death and an ITM and weakening the immune response to the tumor.^[^ ^32^, ^33^ ^]^ During phagocytosis, MerTK on the surface of macrophages recognizes the exposed PtdSer on apoptotic cells through the bridging molecules Gas6 and Protein S, mediating the rapid clearance of apoptotic cells by macrophages.^[^ ^32^, ^34^ ^]^ Since they effectively block PtdSer on the surface of apoptotic tumor cells, decoy EVs may also be able to inhibit macrophage phagocytosis. Therefore, bone marrow‐derived macrophages (BMDMs) were pretreated with resting EVs or decoy EVs at the same concentration for 24 h, followed by coincubation with apoptotic 4T1 cells for 4 h. As expected, decoy EVs significantly reduced the extent of macrophage phagocytosis (Figure 1M,N).

In summary, the above results demonstrate that decoy EVs block ICs to modulate the functions of both T cells and macrophages, making them ideal agents and delivery vehicles for overcoming resistance to immunotherapy.

### 2.2 siTEAD4/MOF Catalyzes Sustainable ROS Production and Mediates TEAD4 Downregulation to Kill Both CSCs and Non‐CSCs

**Figure 2:**
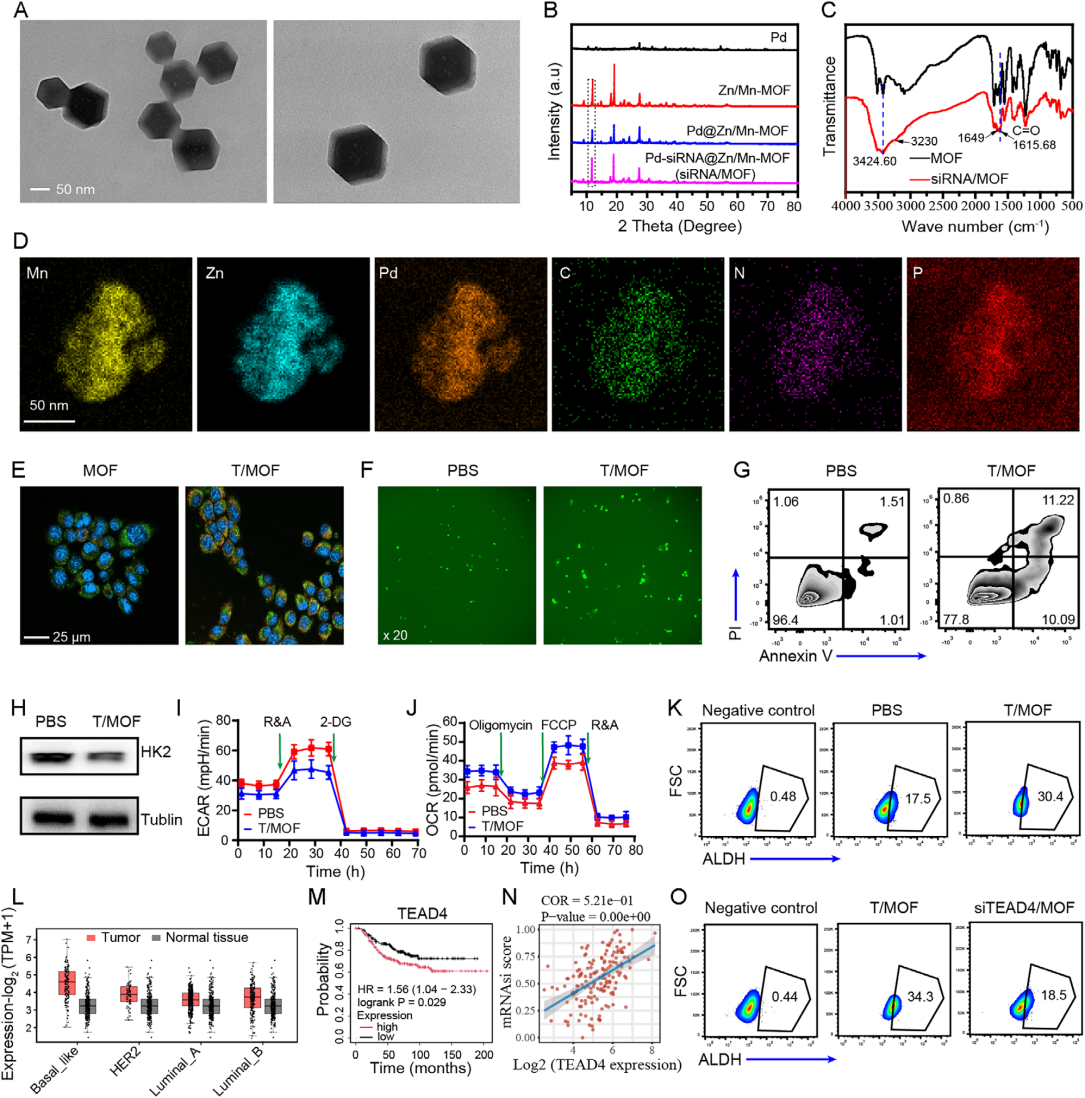
Preparation and characterization of T/MOF. A) TEM images of T/MOF. B) XRD patterns of T/MOF. C) FT‐IR spectra of T/MOF before and after siRNA adsorption. D) Elemental mapping of T/MOF. E) CLSM images of the mitochondrial localization of MOF and T/MOF (blue: DAPI; green: MitoTracker; red: MOF or T/MOF). F) Fluorescence microscopy images of ROS in 4T1 cells treated with PBS or T/MOF for 48 h. G) Analysis of 4T1 cell apoptosis after treatment with PBS or T/MOF for 48 h. H) HK2 expression in 4T1 cells. I) ECAR profiles of 4T1 cells treated with PBS or T/MOF at various time points followed by consecutive injections of rotenone and antimycin A (R&A) or 2‐deoxy‐D‐glucose (2‐DG). J) OCR profiles of 4T1 cells treated with PBS or T/MOF at various time points, followed by consecutive injections of oligomycin, FCCP, or R&A. K) Flow cytometry analysis of ALDH^+^ 4T1 cells. L) Bioinformatics analysis of TEAD4 expression in TNBC cells. M) Kaplan‒Meier survival analysis of TNBC patients with high or low TEAD4 expression.N) Spearman's correlation analysis of TEAD4 levels and TNBC cell stemness. O) Flow cytometry analysis of CSCs (ALDH^+^) among 4T1 cells treated with T/MOF or siTEAD4@EVs for 48 h. The data are presented as the means ± SD (n = 3).

TNBC is highly heterogeneous. Therefore, extensive tumor cell killing is difficult to achieve with IC therapy alone.^[^ ^35^ ^]^ Nanozyme‐mediated CDT can not only directly and specifically kill tumor cells but also trigger ICD in tumors to increase the efficacy of immunotherapy.^[^ ^36^, ^37^ ^]^ In this study, a tetraphenylporphyrin (TPP)‐modified Zn/Mn‐MOF‐encapsulated monoatomic Pd nanozyme (T/MOF) was prepared to target mitochondria. As shown in **Figure** **2**A, the TEM images of T/MOF reveal a unique spindle shape and highly uniform size. The X‐ray diffraction (XRD) patterns revealed that T/MOF has a regular crystalline structure, and after TPP modification and siRNA loading, the crystal phase of the MOF did not change (Figure 2B). In the Fourier transform infrared (FT‐IR) spectrum of the siRNA‐adsorbed MOF (siR/MOF) (Figure 2C), the peak at 1615.68 cm^−1^ was assigned to the C═O stretching vibrations of the ‐CHO group. RNA‐specific vibration peaks at 1649 and 3230 cm^−1^ were also observed in the spectrum of siRNA/MOF, confirming the loading of RNA onto the T/MOF. The XRD mapping images revealed a uniform distribution of Mn, Zn, Pd, C, N, and P in the interior and on the surface of siRNA/MOF (Figure 2D). X‐ray photoelectron spectroscopy (XPS) confirmed the presence of the above elements (Figure S2, Supporting Information). T/MOF decomposition generates O_2_, and the monoatomic Pd nanozyme continuously catalyzes the conversion of O_2_ into the Fenton‐like reaction substrate H_2_O_2_,^[^ ^38^, ^39^ ^]^ leading to O_2_ and H_2_O_2_ self‐cycling. Moreover, Mn^2+^ catalyzes the conversion of H_2_O_2_ into ROS via a Fenton‐like reaction to kill tumor cells. Next, the biological functions of T/MOF were investigated. Confocal laser scanning microscopy (CLSM) revealed significantly enhanced mitochondrial targeting after TPP modification (Figure 2E). The ROS level in 4T1 cells treated with T/MOF also significantly increased (Figure 2F). T/MOF significantly induced apoptosis in 4T1 cells (Figure 2G). To assess the safety of T/MOF, human liver LO2 cells were treated with different concentrations of T/MOF for 48 h. The results revealed that the viability of LO2 cells still exceeded 85% in the high‐dose T/MOF group (100 µg mL^−1^), demonstrating the good biocompatibility of T/MOF (Figure S3, Supporting Information). Additionally, Zn^2+^ has been reported to inhibit hexokinase II (HK2) to restrict aerobic glycolysis in tumors, further promoting antitumor immunity.^[^ ^40^ ^]^ Therefore, we investigated the effects of T/MOF on HK2 protein expression and mitochondrial metabolism. The data revealed that T/MOF significantly inhibited HK2 expression (Figure 2H). Moreover, T/MOF significantly decreased the extracellular acidification rate (ECAR) in 4T1 cells, whereas the oxygen consumption rate (OCR) significantly increased (Figure 2I,J; Figure S4, Supporting Information), indicating that T/MOF effectively inhibits tumor cell glycolysis, which may help reshape the tumor immune microenvironment by decreasing lactate levels and causing energy depletion. However, we found that the T/MOF‐treated group had a larger population of cells positive for ALDH1 (a key marker of CSCs) and increased OXPHOS. ALDH1 can promote the clearance of ROS, and CSCs can rely on OXPHOS for survival. These findings suggest that CSCs are resistant to glycolysis inhibition and high levels of ROS (Figure 2K). CSCs are recognized as important factors leading to the failure of cancer therapy. Therefore, to maximize antitumor efficiency, introducing a CSC inhibitor is necessary. TEAD4 is a key transcription factor downstream of the Hippo signaling pathway that is closely associated with epithelial‒mesenchymal transition (EMT), tumor cell stemness, and immune escape and is an important tumor driver gene. Bioinformatics data revealed that TEAD4 is upregulated in all subtypes of breast cancer, especially TNBC, and patients with high TEAD4 expression have significantly shorter survival times (Figure 2L,M). Furthermore, the data revealed a positive correlation between TEAD4 expression and stemness in TNBC cells (Figure 2N). We subsequently used siTEAD4 to downregulate TEAD4. Notably, siTEAD4 significantly inhibited the migration and invasion of 4T1 cells (Figure S5, Supporting Information). We examined the death of both CSCs and non‐CSCs following TEAD4 knockdown. The results revealed that TEAD4 depletion induced significant death in both CSCs and their non‐CSC counterparts, suggesting its key role in decreasing the survival of CSC and non‐CSC tumor cells (Figure S6, Supporting Information). In addition, consistent with the bioinformatics analysis, siTEAD4 reduced the proportion of CSCs (ALDH^+^) among the 4T1 cells (Figure 2O). These results demonstrate that T/MOF generates excessive ROS and inhibits aerobic glycolysis, whereas siTEAD4 inhibits stemness, thus exerting synergistic antitumor effects.

### 2.3 siT/MOF@EVs Inhibit TNBC Cell Glycolysis/OXPHOS and Trigger ICD Simultaneously

**Figure 3:**
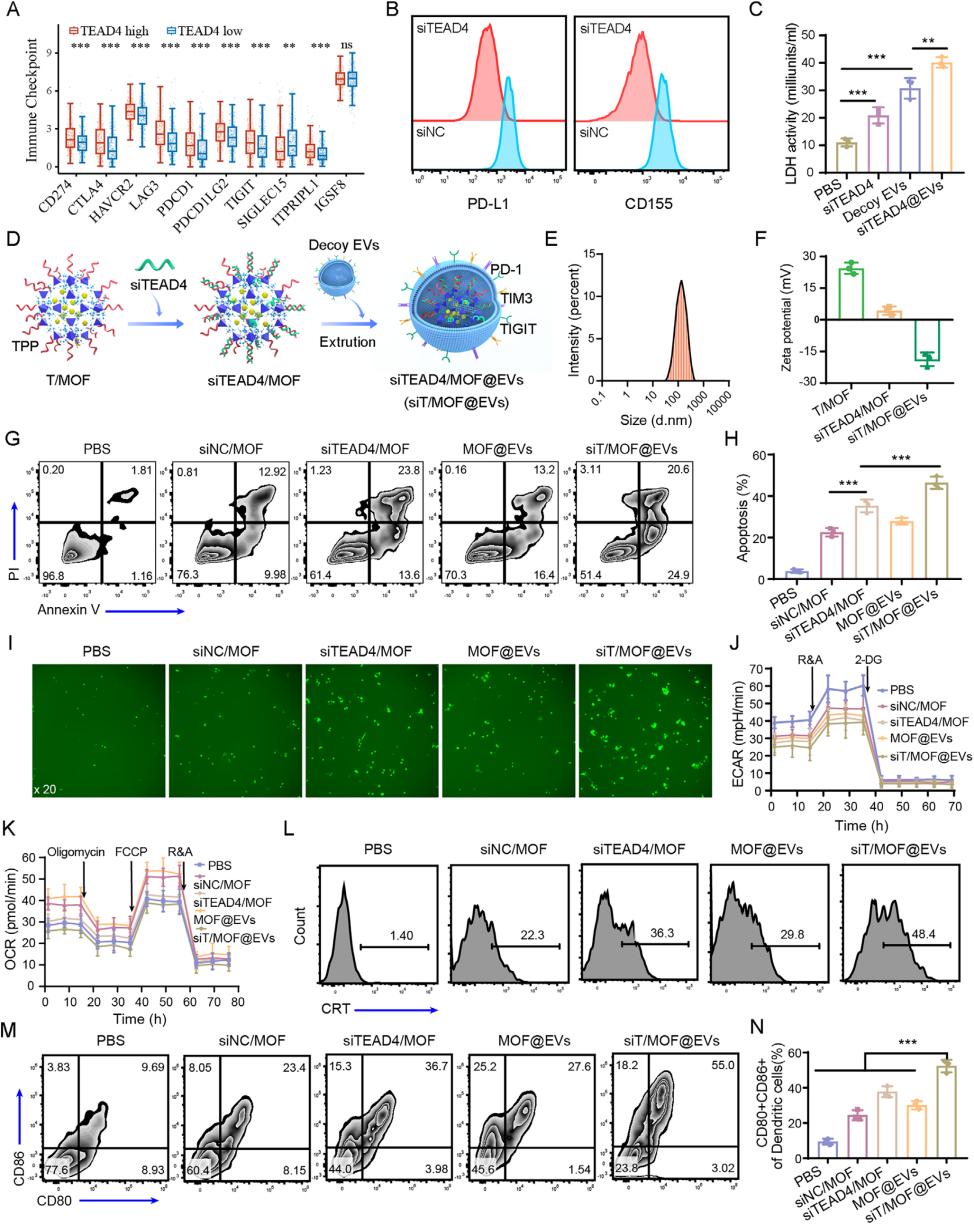
Preparation and characterization of siT/MOF@EVs and evaluation of their antitumor effects in vitro. A) Correlation between TEAD4 expression and the expression of checkpoint‐related genes in TNBC. B) PD‐L1 and CD155 expression on the surface of 4T1 cells treated with siNC or siTEAD4 for 48 h. C) 4T1 cells were cocultured with activated OT‐1 CD8^+^ T cells and treated with PBS, siTEAD4, decoy EVs, or siTEAD4@EVs in the presence of 8 µm OVA peptide for 48 h, and the LDH concentration was measured to evaluate the immune‐mediated killing ability of CD8^+^ T cells. D) Schematic of siT/MOF@EVs preparation. E) Particle size distribution of siT/MOF@EVs. F) Zeta potentials of T/MOF, siTEAD4/MOF, and siT/MOF@EVs. G) Flow cytometry analysis of apoptotic 4T1 cells (Annexin V^+^ *P*I^+^) treated as indicated for 48 h. H) Quantification of the data from panel G. I) Fluorescence microscopy images of ROS in 4T1 cells. J) ECAR profiles of 4T1 cells treated as indicated. K) OCR profiles of 4T1 cells treated as indicated. L) Flow cytometry analysis of the proportion of CRT^+^ 4T1 cells. M) 4T1 cells were pretreated as indicated for 48 h and then cocultured with BMDCs for 24 h. Flow cytometry analysis of mature DCs (CD80^+^CD86^+^). N) Quantification of the data from panel M. The data are presented as the means ± SD (n = 3). p‐values are calculated using one‐way ANOVA with Tukey's multiple comparison. ^**^ *p* < 0.01, ^***^ *p* < 0.001.

To evaluate the antitumor immune effect of siTEAD4 in the context of TNBC, we analyzed the correlation between siTEAD4 and the expression of ICs through bioinformatics analysis. The data revealed that TEAD4 expression was positively correlated with the mRNA levels of multiple ICs (**Figure** **3**A). We subsequently knocked down TEAD4 expression with siTEAD4 and analyzed both the expression of major IC ligands on the surface of 4T1 cells and the toxicity of CD8^+^ T cells to tumor cells. The data revealed that siTEAD4 inhibited the expression of PD‐L1 and CD155 (Figure 3B). More importantly, compared with decoy EVs alone, decoy EVs loaded with siTEAD4 (siTEAD4@EVs) significantly increased the toxicity of CD8^+^ T cells to 4T1 cells (Figure 3C). These results suggest that siTEAD4 can synergize with decoy EVs‐mediated blockade of multiple ICs to exert antitumor immune effects. Therefore, the mitochondrion‐targeted T/MOF was used as the primary carrier onto which siTEAD4 was electrostatically adsorbed before it encapsulated decoy EVs, constructing a delivery system that targets the cell membrane‒cytoplasm‒mitochondria cascade (siT/MOF@EVs) (Figure 3D). The average size of the siT/MOF@EVs was 131.47 ± 8.46 nm, with a polydispersity index (PDI) of 0.17 ± 0.04, and the zeta potential was −17.45 ± 3.02 mV (Figure 3E,F). The siRNA encapsulation efficiency (EE%) of siT/MOF@EVs was 87.3%. Furthermore, agarose gel electrophoresis confirmed the exceptionally high siRNA loading efficiency of the siT/MOF@EVs (Figure S7A, Supporting Information). The size of the siT/MOF@EVs did not significantly change during one week of storage at different temperatures (4, 25, and 37°C), indicating good stability. In addition, siTEAD4 was released from siT/MOF@EVs in a controlled manner (Figure S7B, C, Supporting Information). TEM demonstrated that the siT/MOF@EVs had a uniform particle size and were completely coated with vesicular structures (Figure S8, Supporting Information). Next, Cy5‐siRNA (Cy5‐siR) was used as a fluorescent probe and loaded onto the EVs instead of siTEAD4 to assess the transfection efficiency of siT/MOF@EVs. CLSM revealed that the fluorescence intensity of Cy5‐siR in the siT/MOF@EVs group was significantly greater than that in the siR/MOF group, mainly because of the active targeting of decoy EVs to tumor cells. siT/MOF@EVs also significantly downregulated TEAD4 (Figure S9, Supporting Information). Upon investigating the endocytic mechanisms governing siT/MOF@EVs internalization by tumor cells, quantitative analysis revealed that PitStop 2 treatment caused the most pronounced suppression of siT/MOF@EVs uptake (Figure S10, Supporting Information), strongly suggesting that clathrin‐mediated endocytosis was the predominant entry route. We subsequently investigated the intracellular trafficking and lysosomal escape of the siT/MOF@EVs. The data revealed that the siT/MOF@EVs were internalized within 4 h, colocalized with lysosomes, and escaped from the lysosomes within 8 h, indicating the ability of the siT/MOF@EVs to escape lysosomal degradation (Figure S11, Supporting Information). Moreover, the percentage of apoptotic cells in the siT/MOF@EVs group reached 45.5%, which was significantly greater than that in the other treatment groups, indicating excellent antitumor efficacy (Figure 3G,H). Similarly, the ROS levels in the siTEAD4/MOF, MOF@EVs, and siT/MOF@EVs groups were significantly increased, further confirming that T/MOF can efficiently catalyze ROS production to kill tumor cells (Figure 3I). TEAD4 not only functions as a DNA anchor protein for YAP in the Hippo pathway but also regulates mitochondrial function independently of YAP.^[^ ^14^ ^]^ Research has shown that TEAD4 directly affects OXPHOS activity by regulating the expression of genes encoding enzymes in the mitochondrial–nuclear ETC.^[^ ^17^ ^]^ Thus, the ECAR and OCR were measured to verify the effects of siT/MOF@EVs on cellular glycolysis and OXPHOS. Seahorse assays revealed that siTEAD4 effectively overcame the feedback‐mediated upregulation caused by T/MOF‐mediated glycolysis inhibition. siT/MOF@EVs also inhibited both cellular glycolysis and OXPHOS simultaneously, leading to energy depletion in tumor cells (Figure 3J,K; Figure S12, Supporting Information). To determine whether the oxidative burst generated by siT/MOF@EVs could induce ICD in tumor cells, the expression of the ICD marker calreticulin (CRT) was detected. Compared with that in the PBS group, the expression of CRT was significantly increased in all the treatment groups (Figure 3L, Figure S13, Supporting Information). Moreover, the expression of CRT in the siT/MOF@EVs group was greater than that in the siTEAD4/MOF group, possibly because the active targeting of decoy EVs increased T/MOF uptake by tumor cells. ICD causes the release of DAMPs, which activate DCs to induce tumor‐specific immune responses.^[^ ^41^ ^]^ Therefore, we evaluated whether different treatments promoted DC maturation in 4T1 cells. Consistently, the proportion of mature DCs (CD80^+^CD86^+^) was greater in the siT/MOF@EVs group than in the other treatment groups (Figure 3M,N).

Collectively, these results demonstrate that siT/MOF@EVs exhibit a robust antitumor immune response through intracellular and extracellular positive feedback mechanisms: active targeting by decoy EVs promotes siTEAD4/MOF nanozyme uptake by tumor cells, whereas siTEAD4/MOF‐triggered ICD enhances the immune effects of decoy EVs.

### 2.4 siT/MOF@EVs Suppress Tumor Growth and Spontaneous Pulmonary Metastasis in an Orthotopic TNBC Mouse Model

**Figure 4:**
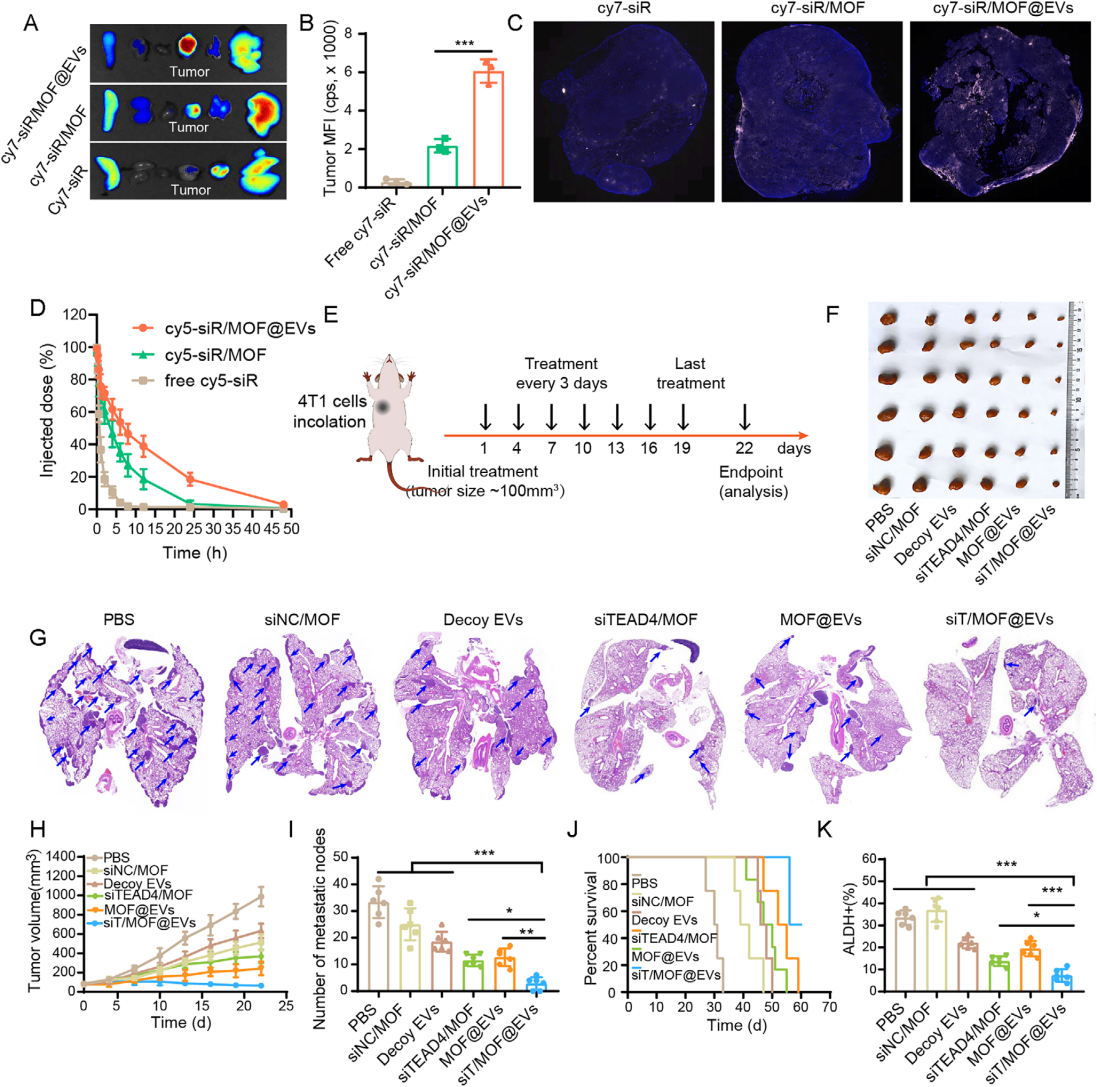
Therapeutic efficiency of siT/MOF@EVs in an orthotopic TNBC mouse model. A) Ex vivo fluorescence images of vital organs and tumors 48 h after injection. B) Fluorescence quantification of the tumor tissue in panel A. C) Fluorescence distribution in tumor tissue. D) Pharmacokinetic profiles of Cy5‐siR, Cy5‐siR/MOF and Cy5‐siR/MOF@EVs. E) Treatment schedule for orthotopic 4T1 tumor‐bearing mice. F) Ex vivo tumor images. G) Images of H&E‐stained lung tissues. The spontaneous pulmonary metastatic nodules were counted. H) Tumor growth curves. I) Number of spontaneous pulmonary metastatic nodules. J) Survival curves of the mice. (K) Flow cytometry analysis of CSCs (ALDH^+^) in 4T1 tumor tissues. The data are presented as the means ± SD (n = 6). p‐values are calculated using one‐way ANOVA with Tukey's multiple comparison. ^*^ *p* < 0.05, ^**^ *p* < 0.01, ^***^ *p* < 0.001.

EVs, as endogenous nanocarriers, combine the advantages of cell‐based drug delivery and nanotechnology: low immunogenicity, penetration of physiological barriers, and both passive and natural active targeting.^[^ ^42^ ^]^ To evaluate the in vivo tumor‐targeting ability of siT/MOF@EVs, Cy7‐siRNA (Cy7‐siR) was used in place of siTEAD4 as a tracer to observe their biodistribution. In vivo fluorescence imaging revealed that Cy7‐siR/MOF@EVs clearly accumulated at the tumor site 2 h after injection and that the fluorescence signal was not significantly attenuated after 48 h (Figure S14, Supporting Information). Similarly, ex vivo fluorescence imaging of tumors and major organs confirmed the preferential accumulation of Cy7‐siR/MOF@EVs in tumor tissues. In addition, the fluorescence intensities of the tumors in the Cy7‐siR/MOF@EVs group were significantly greater than those in the Cy7‐siR/MOF group, suggesting that decoy EVs increased tumor targeting (**Figure** **4**A,B). The increased interstitial fluid pressure and a dense extracellular matrix in solid tumors hinder drug penetration and therapeutic efficacy.^[^ ^43^ ^]^ While decoy EVs have a typical phospholipid bilayer membrane structure and low immunogenicity, their high expression of IC molecules may facilitate siT/MOF@EVs penetration into tumor tissues. The results revealed that the fluorescence signal in the tumors in the free Cy7‐siR group was very weak, which is consistent with the images captured by the fluorescence imaging apparatus. In the Cy7‐siR/MOF group, the fluorescence signal was enriched mainly at the edge of the tumor. In contrast, in the siT/MOF@EVs group, strong fluorescence signals were observed both in the center and periphery of the tumor (Figure 4C), which indicated that siT/MOF@EVs have excellent intratumor‐targeting ability. In addition, pharmacokinetic analysis revealed that the half‐life of the siRNA was significantly prolonged in the siT/MOF@EVs group (Figure 4D), suggesting that the low immunogenicity of EVs endows the siT/MOF@EVs with a long circulation time. The therapeutic efficiency of siT/MOF@EVs was subsequently evaluated in an orthotopic 4T1 tumor mouse model. The tumor volume in the decoy EVs group was significantly smaller than that in the PBS group, suggesting that decoy EVs induce potent and specific T‐cell antitumor immunity via the blockade of multiple ICs in vivo. Importantly, siT/MOF@EVs exhibited significantly greater therapeutic efficacy than the other treatments and significantly suppressed spontaneous lung metastasis, demonstrating their good synergistic antitumor effect (Figure 4E–I). As shown in Figure 4J, siT/MOF@EVs treatment significantly prolonged the survival time of the model mice. Eliminating CSCs is an important strategy to overcome drug resistance, and in vitro data also revealed that siTEAD4 could inhibit the stemness of TNBC cells. Flow cytometry was performed to analyze the ratio of CSCs to total cells. The data revealed that the proportion of CSCs (ALDH^+^) in the siTEAD4 treatment groups was decreased (Figure 4K). Furthermore, the proportion of CSCs in the siT/MOF@EVs group was the lowest, which may be related to the increased intratumoral penetration of siTEAD4 and multi‐IC blockade mediated by the decoy EVs. The target genes of YAP/TEAD4 (CTGF and CYR61) were significantly downregulated in the siTEAD4/MOF‐ and siT/MOF@EVs‐treated groups (Figure S15, Supporting Information), indicating that the YAP/TEAD4 pathway was suppressed after TEAD4 downregulation. To evaluate the inhibitory effects of the combination of siTEAD4 and T/MOF on glycolysis and OXPHOS, the key genes involved in these processes were detected by qPCR. The results demonstrated that siT/MOF@EVs treatment significantly suppressed both glycolysis and OXPHOS in tumor cells in vivo (Figure S16, Supporting Information). To evaluate changes in ROS levels in tumor cells under different treatment conditions, tumor tissues were isolated and dissociated into single‐cell suspensions. The cells were then stained with DCFH‐DA for 20 min, washed, and subjected to flow cytometry analysis. The results showed that MOF‐containing nanoparticle (NP) treatment significantly increased the ROS level, indicating that MOF triggers an oxidative burst (Figure S17, Supporting Information).

With respect to the biosafety of siT/MOF@EVs, the body weights of the mice in the siT/MOF@EVs group were not obviously lower than those in the PBS group. Liver (ALT and AST) and kidney function (CREA) indicators were also within normal ranges (Figure S18, Supporting Information). Similarly, no significant pathological changes were observed in the major organs of the mice treated with siT/MOF@EVs (Figure S19, Supporting Information). These results demonstrate that siT/MOF@EVs do not have significant systemic toxicity.

### 2.5 siT/MOF@EVs Reverse the Immunosuppressive Tumor Microenvironment and Induce Protective Immune Memory

Research has shown that ICs such as PD‐1 and TIM‐3 have critical inhibitory effects on T cell, macrophage, and DC activation.^[^ ^44^ ^]^ Therefore, blocking multiple ICs simultaneously is beneficial for synergistically overcoming tumor immune escape. Furthermore, ICD acts as an in situ vaccine by inducing DAMP release, activating innate immune cells, and promoting tumor immune circulation. More importantly, CSCs possess immune escape properties and can shape an immunosuppressive microenvironment, which is crucial for tumor immune resistance and recurrence. The decoy EVs developed here can simultaneously block multiple ICs and preferentially target CSCs to deliver siTEAD4/MOF. siTEAD4/MOF can downregulate multiple ICs at the transcription level and effectively kill CSCs, thus mediating ICD. We further analyzed the immune mechanisms underlying these therapeutic effects.

**Figure 5:**
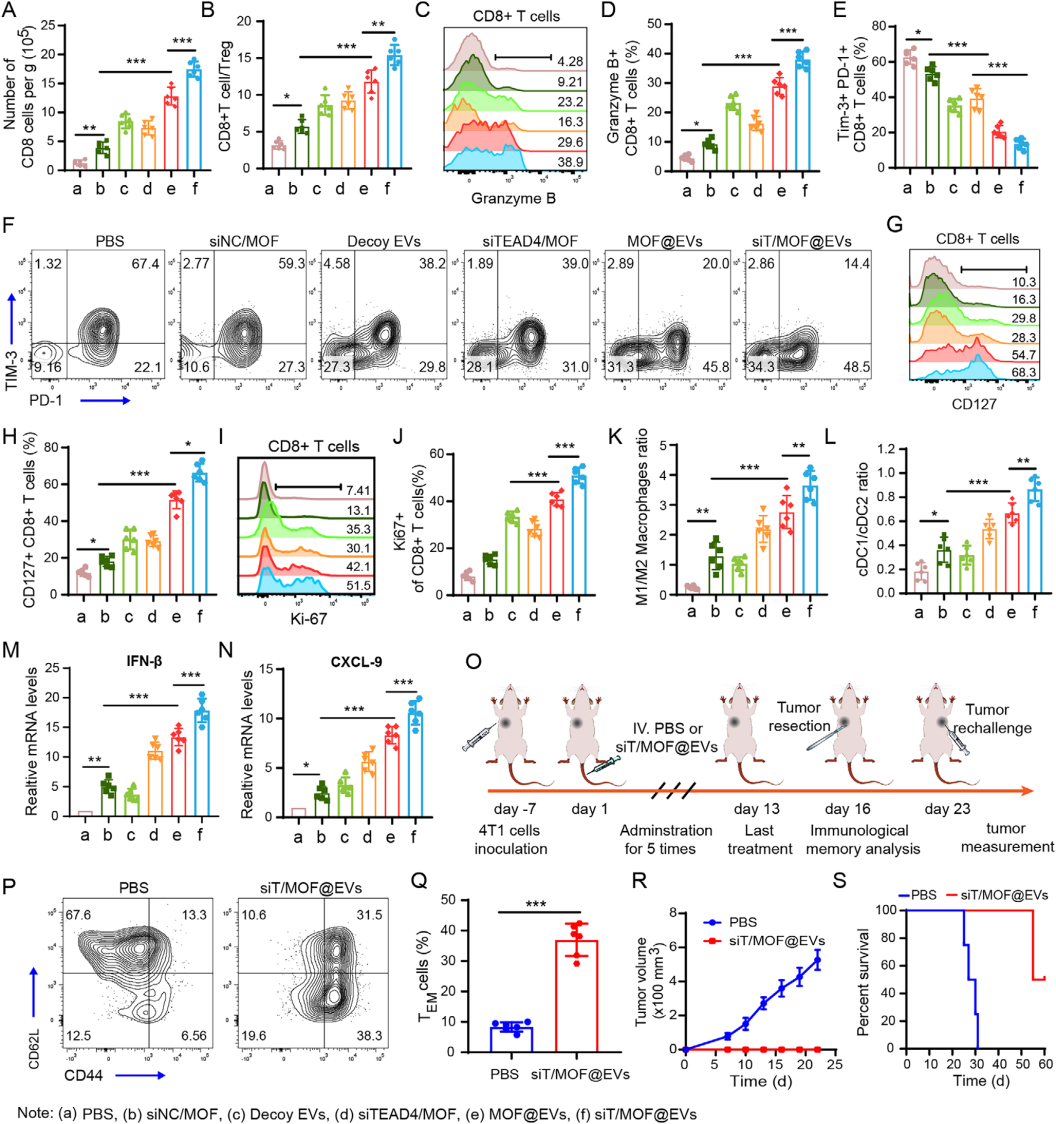
Immune microenvironment regulation and protective antitumor immune memory effects of siT/MOF@EVs. A–L) At the treatment endpoint, TILs were isolated for flow cytometry analysis. (A) Number of CD8^+^ T cells per gram of tumor tissue. (B) Ratio of CD8^+^ T cells/Treg cells. (C) Flow cytometry plots of Granzyme B^+^ cells among CD8^+^ TILs. (D) Statistical analysis of the data from panel C. (E) Statistical analysis of exhausted CD8^+^ TILs. (F) Flow cytometry plots of exhausted CD8^+^ TILs (PD‐1^+^TIM‐3^+^). (G) Flow cytometry plots of Ki‐67^+^ cells among CD8^+^ TILs. (H) Statistical analysis of the data from panel E. (I) Flow cytometry plots of CD127^+^ cells among CD8^+^ TILs. (J) Statistical analysis of the data from panel I. (K) Ratio of cDC1/cDC2 cells. (L) Ratio of M1/M2 macrophages. M, N) mRNA levels of IFN‐β (M) and CXCL9 (N) in tumor tissue detected by qPCR. O) Schematic of the immune memory assay. 4T1 tumor‐bearing mice treated with PBS or siT/MOF@EVs were divided into three groups. Splenic lymphocytes were isolated from 1/3 of the mice (n=6) for flow cytometry analysis. The primary tumors of the other mice were excised, and the mice were reinoculated with 4T1 cells on the contralateral side one week later. The growth of the second tumor (n=6) and survival time of the mice (n=6) were recorded. P) Splenic T_EM_ cells (CD44^high^CD62^low^) analyzed by flow cytometry. Q) Statistical analysis of the data from panel P. R) Growth curves of the second tumors. S) Survival time of the mice after rechallenge with 4T1 tumors. The data are presented as the means ± SDs (n = 6). p‐values are calculated using one‐way ANOVA with Tukey's multiple comparison. ^*^ *p* < 0.05, ^**^ *p* < 0.01, ^***^ *p* < 0.001.

The gating strategy for immune cells is shown in Figures S20 and S21 (Supporting Information). We found that the number of CD8^+^ tumor‐infiltrating lymphocytes (TILs) and the CD8^+^ T‐cell/Treg cell ratio were significantly greater in all the treated groups than in the control group (**Figure** **5**A,B). Moreover, the number of granzyme B (GB)^+^ cells was significantly greater in the siT/MOF@EVs group (Figure 5C,D). However, the siNC/MOF treatment did not reverse T‐cell exhaustion, whereas the decoy EVs, siTEAD4/MOF, and MOF@EVs groups presented significantly fewer exhausted CD8^+^ T cells (PD‐1^+^TIM‐3^+^). In addition, the siT/MOF@EVs group had the lowest percentage of exhausted CD8^+^ T cells (Figure 5E,F). PD‐1^+^TIM‐3^‐^ CD8^+^ T cells contain both stem‐like and effector‐like TILs, which are crucial for long‐lasting antitumor immunity. We found that the highest expression of both the memory marker CD127 and the effector lineage marker Ki‐67 was in CD8^+^ TILs, indicating that siT/MOF@EVs could best support the maintenance and proliferation of stem‐like and effector CD8^+^ TILs (Figure 5G–J). STING activation increases the expression of interferon‐stimulated genes (ISGs), which promote innate immune cell activation and antigen presentation. We found a significant increase in the expression of ISGs and the number of conventional type 1 DCs (cDC1s) in the siT/MOF@EVs group (Figure 5K‐M). Furthermore, M2 tumor‐associated macrophages (TAMs) in the TME are crucial for maintaining the immunosuppressive microenvironment and stem cells. We found that the M1/M2 macrophage ratio was significantly increased in the siT/MOF@EVs group, indicating that these EVs can reverse the immunosuppressive effects of TAMs (Figure 5N). We propose that siT/MOF@EVs block multiple ICs and induce ICD and CSC killing to reverse the immunosuppressive microenvironment and achieve the best antitumor immune activation effect. To further examine whether siT/MOF@EVs could enhance tumor‐specific immune memory, we conducted a tumor rechallenge experiment, as illustrated in Figure 5O. First, we examined spleen cells from tumor‐bearing mice and found that the proportion of effector memory T (T_EM_) cells (CD44^high^CD62^low^) was significantly greater in siT/MOF@EVs‐treated mice than in control mice (Figure 5P,Q). The tumor volume and survival curve data revealed that siT/MOF@EVs treatment induced protective immune memory to inhibit tumor recurrence (Figure 5R,S).

### 2.6 siT/MOF@EVs Significantly Inhibited Tumor Growth in a Humanized PDOX Mouse Model

**Figure 6:**
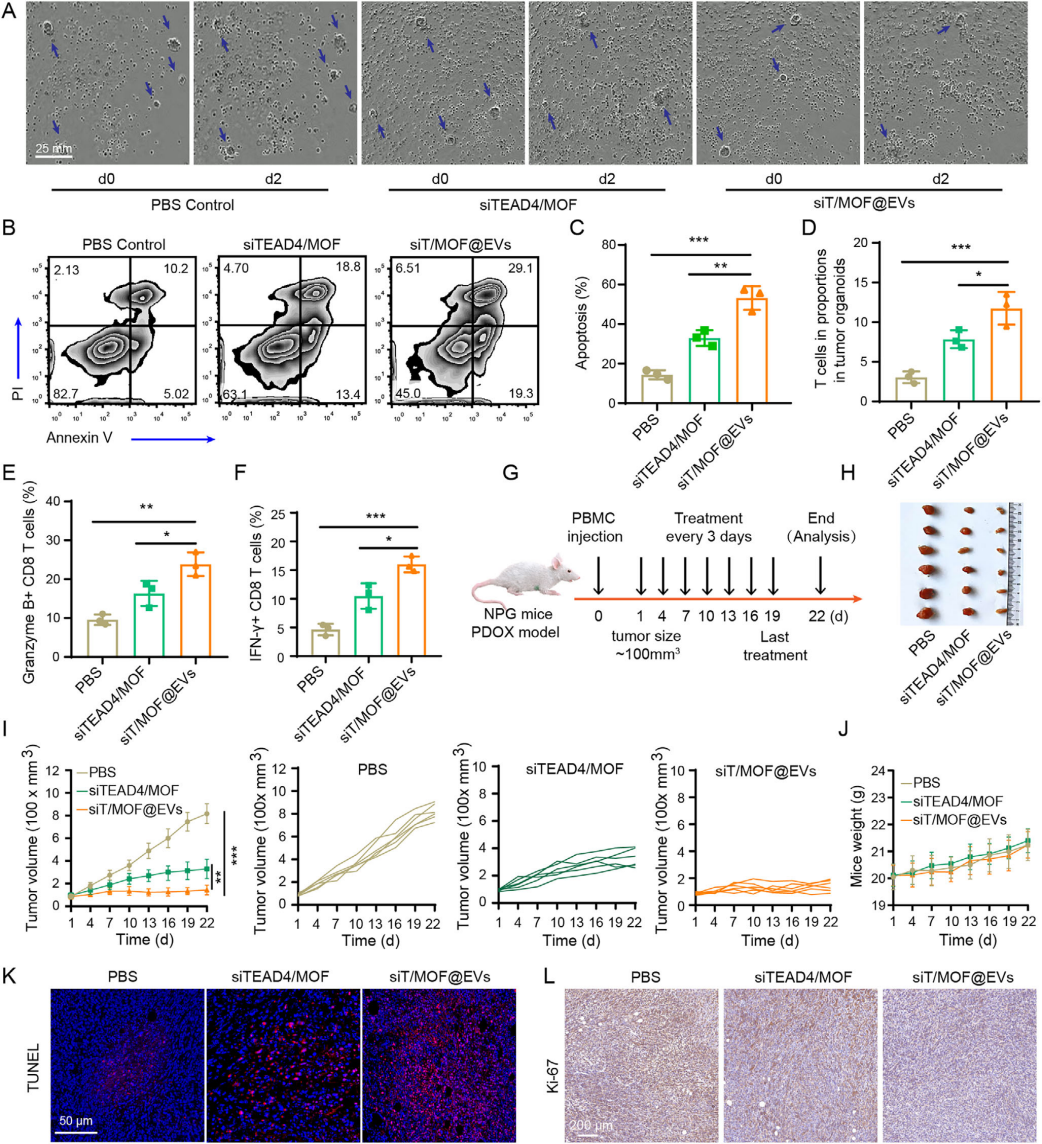
Antitumor efficacy of siT/MOF@EVs on TNBC PDOs in vitro and in vivo. A–F) TNBC PDOs were cocultured with activated T cells treated with PBS, siTEAD4/MOF, or siT/MOF@EVs for 48 h. Images of CD8^+^ T cells killing PDOs (A). Flow cytometry analysis of tumor cell apoptosis in PDOs (B). Quantification of PDO‐induced tumor cell apoptosis (C). Proportions of infiltrating T cells in the organoids (D). Proportions of granzyme B^+^ cells among CD8^+^ T cells (E). Proportions of IFN‐γ^+^ cells among CD8^+^ T cells (F). G) Experimental schedule of siT/MOF@EV treatment in the PBMC humanized PDOX model. H) *Ex vivo* tumor images. I, J) Curves of the changes in tumor volume (I) and mouse weight (J). (J) TUNEL‐stained images of PDOX‐induced tumor cell apoptosis. K) Immunohistochemical staining images of Ki‐67 expression in PDOX tumor tissues. Data are presented as the mean ± SD (For in vitro assays (C–F), n = 3. For in vivo assays (I,J), n = 6). p‐values are calculated using one‐way ANOVA with Tukey's multiple comparison. ^*^ *p* < 0.05, ^**^ *p* < 0.01, ^***^ *p* < 0.001.

Patient‐derived organoids (PDOs) retain most of the characteristics of their primary tumors in terms of histopathology, biomarkers, and genetic features, demonstrating good clinical relevance.^[^ ^45^ ^]^ Furthermore, PDOs are derived from cells with stem cell potential, making them excellent models for evaluating the suppression of tumor stemness by siT/MOF@EVs. In this study, TNBC PDOs were prepared, and the expression of TEAD4 in these organoids and adjacent cancer tissues was detected. The results revealed that TEAD4 was significantly upregulated in the organoids (Figure S22, Supporting Information). To evaluate the antitumor immune effects of siT/MOF@EVs, CD3^+^ T cells (purity ≥95%) were isolated from PBMCs and preactivated with αCD3/αCD28. PDOs were subsequently cocultured with preactivated T cells with PBS (control), siTEAD4/MOF, or siT/MOF@EVs for 2 days, after which the morphology of the PDOs was observed. The results revealed that T cells aggregated around and infiltrated the PDOs, with greater aggregation of T cells within the PDO region in the siT/MOF@EVs group. Moreover, in the PBS group, the morphology of most PDOs did not significantly change, whereas in the siTEAD4/MOF and siT/MOF@EVs groups, the PDOs were smaller and had partially disintegrated, with a more pronounced change in the siT/MOF@EVs group (**Figure** **6**A).

Next, the PDOs were digested to generate single‐cell suspensions, and tumor cell apoptosis and T‐cell infiltration and function were analyzed by flow cytometry. The data revealed that after siT/MOF@EVs treatment, the organoid apoptosis rate reached 48.4%, which was significantly greater than that after PBS (15.22%) and siTEAD4/MOF treatment (32.2%) (Figure 6B,C). Additionally, the data showed significantly more infiltrating T cells within the PDOs in the siT/MOF@EVs group (Figure 6D). Similarly, siT/MOF@EVs significantly increased the levels of the key cytotoxicity factors IFN‐γ and Granzyme B in the CD8^+^ T cells infiltrating the organoids (Figure 6E,F), indicating that siT/MOF@EVs can better promote the antitumor immune function of CD8^+^ T cells.

PDOX models can maintain tumor heterogeneity and the immune microenvironment to better mimic the characteristics of the parental tumor.^[^ ^46^ ^]^ The clinical effect of a drug can be predicted via a PDOX model before the treatment is applied to patients. Therefore, PDOs were transplanted into NOD/Prkdcscid/IL‐2Rγnull (NPG) mice to generate a PDOX model. Then, PBMCs were injected to reconstitute the human immune system, and the antitumor effect of siT/MOF@EVs was evaluated (Figure 6G). As expected, the administration of siT/MOF@EVs significantly inhibited tumor growth in the PBMC‐PDOX model (Figure 6H,I). In addition, the body weights of the mice slowly increased, further demonstrating that siT/MOF@EVs have optimal biosafety (Figure 6J). At the end of the treatment, the tumor tissues were sectioned and stained. The TUNEL‐stained images revealed that siT/MOF@EVs efficiently induced apoptosis in PDO tumor cells in vivo (Figure 6K). The tumor proliferation‐associated marker Ki‐67 was detected using immunohistochemistry. Similarly, Ki‐67 was downregulated in PDO tumors treated with siT/MOF@EVs (Figure 6L).

Collectively, these results demonstrated that siT/MOF@EVs overcame the heterogeneity of TNBC without significant systemic toxicity, showing their potential for clinical application.

## 3 Conclusion

In this study, we developed a cascade‐targeted therapeutic platform (siT/MOF@EVs) that sequentially engages tumor membrane receptors, cytoplasmic signaling, and mitochondria. The decoy EVs block ICs (PD‐1/TIM‐3/TIGIT) while simultaneously enabling the tumor‐selective delivery of the siTEAD4/MOF nanocomplex. At the subcellular level, T/MOF induces sustained ROS‐mediated mitochondrial damage and ICD through Pd/Mn catalytic cascades, whereas Zn^2^⁺ suppresses glycolysis via HK2 inhibition. Co‐delivered siTEAD4 silences TEAD4 to suppress CSC maintenance and OXPHOS‐dependent metabolic escape, resulting in energy synthesis disruption.

siT/MOF@EVs effectively overcome TNBC heterogeneity, inhibit tumor growth and metastasis, and induce protective immune memory. In summary, the multimodal siT/MOF@EVs system achieves the synergistic antitumor effects of immunotherapy, siRNA therapy, and chemodynamic therapy while ensuring safety through a tumor‐specific targeting cascade, thus providing a potential strategy to overcome TNBC heterogeneity.

## 4 Experimental Section

### Materials, Cells, and Animals

Zn(NO_3_)_2_·6H_2_, Mn(CH_3_COO)_2_·4H_2_O, 2‐methylimidazole, and Na_2_PdCl_4_ were obtained from Sigma‒Aldrich (St. Louis, USA). Triphenylphosphine was obtained from Aladdin Biochemical Technology Co., Ltd. (Shanghai, China). All siRNAs were purchased from Ruibo Biotechnology, Inc. (Guangzhou, China). The MitoTracker fluorescent probes, ROS assay kits, apoptosis assay kits, and LDH cytotoxicity assay kits were purchased from Beyotime Biotechnology, Inc. (Shanghai, China). cDNA synthesis and qPCR kits were purchased from Vazyme Biotechnology, Inc. (Nanjing, China). IL‐2, IL‐4, and IL‐10 were obtained from PeproTech (Rocky Hill, USA). The sources of the antibodies used in this study are listed in Table S1 (Supporting Information). 4T1 cells (CSTR:19375.09.3101MOUTCM32) were obtained from the Cell Bank of the Chinese Academy of Sciences (Shanghai, China). Female BALB/c mice and SD rats were obtained from Shanghai Slack Laboratory Animals Co., Ltd. (Shanghai, China). NPG mice were obtained from Beijing Vitalstar Biotechnology Co., Ltd. (Beijing, China). The mice were maintained under specific pathogen‐free conditions. The animal experiments were performed under protocols approved by the Animal Care and Use Committee of Wannan Medical College (ethics no. WNMC‐AWE‐2023085).

### Preparation of the T/MOF Nanozyme

First, ultrasonication was used to disperse Zn(NO_3_)_2_·6H_2_O (0.238 g) and Mn(CH_3_COO)_2_·4H_2_O (0.098 g) in 50 mL of methanol to obtain solution A. Then, 2‐methylimidazole (0.81 g) was dissolved by ultrasonication in methanol (50 mL) to obtain a clear solution B. Finally, solution A was poured into solution B with stirring for 45 min. The product was separated by centrifugation and washed with methanol three times. Zn/Mn‐BTC was obtained after drying under vacuum at 60°C.

Subsequently, 100 mg of Zn/Mn‐BTC was dispersed in 5 mL of acetone and stirred for several minutes at room temperature. One milligram of NaPdCl_2_·4H_2_O was dissolved in 5 mL of acetone, this solution was added dropwise to the dispersion of Zn/Mn‐BTC, and the mixture was stirred vigorously at room temperature for 12 h. After the reaction was complete, the product was obtained by centrifugation, washed twice with acetonitrile, and finally dried under vacuum at 25°C overnight. The conjugation of TPP to MOF was achieved by mixing 0.5 mL of TPP solution with 0.5 mL of Zn/Mn‐BTC solution with stirring for 24 h, after which the mixture was lyophilized to obtain TPP‐modified Zn/Mn‐MOF (T/MOF).

### Enrichment of T/MOF in Tumor Mitochondria

4T1 cells were treated with free rhodamine (Rb) or Rb‐labeled T/MOF for 6 h, and then the mitochondria and nuclei were stained with MitoTracker and DAPI, respectively, according to the manufacturer's instructions. Mitochondrial targeting of T/MOFs in 4T1 cells was observed via CLSM (Olympus).

### Bioinformatic Analysis of TEAD4 Expression in TNBC Tissues

The expression of TEAD4 in breast cancer tissues was analyzed using the GEPIA2 tool (http://gepia2.cancer‐pku.cn). The relationship between TEAD4 and survival time in TNBC patients was analyzed with Kaplan‒Meier Plotter (https://kmplot.com/analysis/). Moreover, RNA‐seq data were downloaded from The Cancer Genome Atlas (TCGA). The relationship between TEAD4 expression and TNBC stemness was analyzed via Spearman's correlation analysis. Differences in the mRNA levels of IC‐related genes between TNBC patients with high and low TEAD4 expression were evaluated with the Wilcoxon test.

### Effects of siTEAD4 on the Expression of PD‐L1 and CD155 in 4T1 Cells

4T1 cells were treated with a negative control siRNA (siNC) or siTEAD4 for 48 h and then stained with an anti‐APC‐PD‐L1 antibody and an anti‐FITC‐CD155 antibody for 40 min at 4°C. PD‐L1 and CD155 expression were subsequently analyzed by flow cytometry.

### Effects of siTEAD4 on Antitumor Immunity In Vitro

4T1 cells were cocultured with activated OT‐1 CD8^+^ T cells in 96‐well plates at a tumor cell to‐T‐cell ratio of 1:5 and treated with PBS, siTEAD4 (100 nm), decoy EVs (20 µg mL^−1^), or siTEAD4@EVs in the presence of 8 µm OVA peptide at the indicated time points. An LDH detection kit was used to measure the LDH concentration in the supernatant to evaluate the immune killing effect of CD8^+^ T cells.

### Mechanism of siT/MOF@EVs Cellular Uptake in Vitro

To investigate the mechanism by which siT/MOF@EVs were taken up by tumor cells, the effects of specific inhibitors were evaluated by pretreating the cells for 15 min with 25 µm PitStop 2 (a clathrin‐mediated endocytosis inhibitor), 50 µm EIPA (a macropinocytosis inhibitor), or 2.5 mm MβCD (a cholesterol‐depleting agent that affects lipid raft‐dependent uptake) prior to NP exposure. Following inhibitor treatment, the cells were incubated with the NPs for 6 h before collection and subsequent analysis.

### Isolation of CD8^+^ T‐Cell‐Derived EVs

Ultracentrifugation was performed to extract CD8^+^ T‐cell‐derived EVs. First, the supernatant of cultured T cells was centrifuged at 2000 × g for 30 min to remove cellular debris and at 10,000 × g for 50 min to remove small impurities. Then, the resulting supernatant was filtered through a 0.22 µm filter membrane and centrifuged at 120 000 × g for 110 min to precipitate EVs. Next, purified EVs were obtained by centrifugation at 120 000 × g for 70 min. TEM (Hitachi HT7800, Japan) was used to observe the morphology of the EVs, and NTA (Malvern, UK) was used to measure the particle size and concentration. Western blotting was used to evaluate the expression of EV markers (Alix and Tsg101) and cytoplasmic proteins (GAPDH and Calnexin). The expression of membrane surface ICs was analyzed via flow cytometry.

### PtdSer, CD155 and PD‐L1 BLOCKING Assays

4T1 cells were treated with 20 µg mL^−1^ decoy EVs for 30 min. The cells were then stained with fluorescently labeled anti‐PtdSer, anti‐CD155, and anti‐PD‐L1 antibodies for 40 min. Binding of the antibodies to the IC ligands PtdSer, CD155, and PD‐L1 was detected using flow cytometry.

### In Vitro Efferocytosis Assay

Mouse‐derived bone marrow cells were stimulated with M‐CSF (10 ng mL^−1^) for 1 week to obtain BMDMs. BMDMs were pretreated with resting EVs or decoy EVs for 24 h. 4T1 cells were irradiated with a 254 nm ultraviolet lamp for 30 min to induce apoptosis and labeled with pHrodo™ Red dye (1 mg mL^−1^). The labeled cells were stained again with Annexin V‐APC and then cocultured with the BMDMs at an apoptotic cell to BMDM ratio of 4:1 for 2 h. The efferocytosis of apoptotic 4T1 cells by macrophages was analyzed by flow cytometry.

### Preparation of siT/MOF@EVs

The above extracted EVs were placed in a hypotonic solution containing protease inhibitors at 4°C overnight and then subjected to ultracentrifugation at 120 000 × g for 70 min to obtain EV membranes. The EV membranes were mixed with the same volume of T/MOF solution containing siTEAD4, and siT/MOF@EVs were obtained by repeated extrusion through a liposome extruder containing a 220 nm polycarbonate membrane. The particle size distribution and surface potential were measured by dynamic light scattering (Nano ZS, Malvern).

### Release of siTEAD4 from siT/MOF@EVs

Cy5‐siRNA was used instead of siTEAD4 to prepare siT/MOF@EVs. Two milliliters of siT/MOF@EVs or free Cy5‐siRNA solution was added to a dialysis bag (MW cutoff of 30 kDa). The dialysis bag was then placed in a centrifuge tube containing 18 mL of DEPC water with gentle shaking at 37°C. Two hundred microliters of the external solution was removed at different time points, and the Cy5‐siRNA concentration in the external solution was determined with a fluorescence spectrophotometer (SLM Instruments). The Cy5‐siRNA release curve was plotted.

### Immune Cell Typing by Flow Cytometry

The immune cells were first stained with Zombie Violet™ Fixable Viability dye for 20 min at 4°C to identify dead and live cells. After thorough rinsing, the cells were stained with the corresponding fluorescently labeled antibodies for 40 min at 4°C and washed 3 times with FACS buffer for flow cytometry analysis. For intracellular staining, the cells were stimulated with PMA (50 ng mL^−1^), brefeldin A (10 mg mL^−1^), and ionomycin (750 ng mL^−1^) for 4 h and then permeabilized and fixed before antibody treatment.

### ECAR and OCR Assays

ECAR and OCR assays were performed using a Seahorse XFe96 analyzer (Agilent). 4T1 cells (1×10^4^ cells well^−1^, 80 µL) were seeded in XFe96 cell culture microplates and treated with PBS, siNC/MOF, siTEAD4/MOF, MOF@EVs, or siT/MOF@EVs overnight. The ECAR was measured using a Glycolysis Stress Test Kit under basal conditions. Rot/AA (0.5 µm) and 50 mm 2‐DG were added sequentially. The OCR was measured with a Cell Mito Stress Test Kit under basal conditions. Oligomycin (2.5 µm), FCCP (2 µm), and Rot/AA (0.5 µm) were added sequentially. ECAR and OCR levels were analyzed using Agilent Seahorse Wave Desktop software (version: 2.6.1).

### Apoptosis and Intracellular ROS Assays

4T1 cells were treated with PBS siNC/MOF, siTEAD4/MOF, MOF@EVs, or siT/MOF@EVs (100 nm siRNA, 25 µg mL T/MOF, 20 µg mL^−1^ EVs). For the apoptosis assay, the cells were double stained with Annexin V‐FITC/PI according to the manufacturer's instructions and then analyzed by flow cytometry. For the intracellular ROS assay, the cells were incubated with DCFH‐DA fluorescent probe for 30 min at 37°C and then visualized using a fluorescence microscope (IX71, Olympus).

### In Vitro Analysis of DC Maturation

Before starting BMDC‐4T1 cell cocultures, BMDCs were obtained by stimulating bone marrow cells with GM‐CSF (20 ng mL^−1^) and IL‐4 (10 ng mL^−1^) for 1 week. 4T1 cells were pretreated as indicated (the same treatments as those used for the ROS assay) for 48 h and then cocultured with BMDCs for an additional 24 h. Mature DCs (CD80^+^CD86^+^) were analyzed via flow cytometry.

### Organoid Construction and Culture

Fresh human TNBC tissue was obtained from Yijishan Hospital of Wannan Medical College (ethics no. LLSC‐2022‐63). The tissues were cut into pieces and then digested with a digestive solution containing collagenase for 20 min at 37°C. The digested tissue was subsequently filtered through a 100 µm diameter cell sieve. The collected filtrate was centrifuged, and the cell precipitates were resuspended in a 25‐fold volume of basement membrane extract (BME). The cell mixture was then seeded in a 24‐well plate. When the Matrigel solidified, 500 µL of organoid culture medium (OBM‐500) was added for further culture. The organoids were observed daily under a microscope to evaluate morphology, proliferation rate, microbial contamination, and other parameters. After the organoids had grown to a suitable size, efficacy experiments or digestive passages were carried out.

### Organoid Killing by T Cells

Before organoid–T‐cell coculture, human PBMCs were sorted with a human T‐cell negative isolation kit to obtain purified T cells. The T cells were activated by αCD3/CD28 stimulation for 24 h. TNBC organoids were gently collected from the Matrigel with a pipette and placed in 15 mL centrifuge tubes. After centrifugation for 5 min, the supernatant was discarded, and the organoids were digested for 2–3 min. Digestion was terminated by the addition of organoid passage culture buffer G. After another centrifugation step, the organoids were cocultured with activated T cells and treated with PBS, siTEAD4/MOF, or siT/MOF@EVs for 48 h, after which the morphology of the organoids was observed by microscopy. The organoids were subsequently digested and collected, and organoid tumor cell apoptosis and the infiltration of T cells into the organoids were evaluated. T‐cell reactivity was detected by flow cytometry for Granzyme B and IFN‐γ expression.

### Pharmacokinetic Analysis

SD rats were injected with free Cy5‐siR, Cy5‐siR/MOF, or Cy5‐siR/MOF@ EVs via the tail vein, and 200 µL of blood was collected at 0, 0.25, 0.5, 1, 2, 4, 6, 8, 12, 24, and 48 h after injection to obtain plasma. The plasma concentration of Cy5‐siR in each sample was determined using a fluorescence spectrophotometer (SLM Instruments).

### In Vivo Biodistribution and Intratumoral Penetration

4T1 cells (5×10^5^ cells/100 µL) were inoculated into the 3th mammary fat pad of female BALB/c mice to construct a TNBC orthotopic tumor mouse model. As a tracer, Cy7‐siR replaced siTEAD4 in vivo. Free Cy7‐siR, Cy7‐siR/MOF or Cy7‐siR/MOF@EVs were injected into 4T1 tumor‐bearing mice via the tail vein. The fluorescence distribution in the mice in vivo was dynamically observed using a fluorescence imaging system (Berthold Technologies, Germany). Ex vivo fluorescence images of vital organs and tumors were captured 48 h after injection. For the intratumoral penetration assay, the tumor tissues were made into slices, and the fluorescence distribution within the tumors was observed using an optical microscope.

### Therapeutic Efficacy and Biosafety of siT/MOF@EVs in an Orthotopic 4T1 Tumor Model

When the tumor volume reached ≈100 mm^3^, 4T1 tumor‐bearing mice were *i.v*. injected with PBS, siNC/MOF, decoy EVs (200 µg per mouse), siTEAD4/MOF, MOF@EVs, or siT/MOF@EVs (1.5 mg/kg siTEAD4, 1 mg/kg MOF) every 3 days for a total of 7 injections. The tumor volume, body weight, and survival time of the mice were monitored. At the end of treatment, the plasma concentrations of ALT, AST, and CREA were measured using the corresponding biochemical kits to assess the liver and kidney functions of the mice. The mice were euthanized, and vital organs and tumor tissues were collected for hematoxylin and eosin (H&E) staining. TILs were extracted for flow cytometry analysis. The numbers and functions of T cells, M1/M2 macrophages, and cDC1s/cDC2s were examined to analyze the tumor immune microenvironment.

### Therapeutic Efficacy and Biosafety of siT/MOF@EVs in a PDOX Mouse Model

TNBC organoids were digested and then inoculated into the 4th mammary fat pad of female NPG mice (1× 10^6^ cells per mouse). The mice were *i.v*. injected with human PBMCs (1 × 10^7^/mouse) 1 day before treatment to generate the humanized PBMC‐PDOX model. When the tumor volume reached ≈100 mm^3^, the mice were divided into 3 groups (n = 6): the PBS, siTEAD4/MOF, and siT/MOF@EVs groups. Systemic administration of the indicated agents was performed every 3 days for a total of 7 treatments. Tumor volume and body weight were monitored. The tumor tissues were collected to analyze apoptosis via TUNEL staining and Ki‐67 expression by immunohistochemistry.

### Statistical Analysis

The data were analyzed using GraphPad Prism (version 8.0.1). The data were shown as the means ± SD. The data distributions of values were checked for Gaussian distribution. For normally distributed data, differences between two groups were analyzed by Student's *t*‐test, and differences among multiple groups were analyzed via one‐way ANOVA with Tukey's multiple comparison post‐hoctest. For all the in vitro experiments, n = 3 (3 biologically independent reduplicates). For all the in vivo experiments, n = 6 (6 mice per groups). ^*^ *P* < 0.05, ^**^ *P* < 0.01 and ^***^ *P* < 0.001.

## Conflict of Interest

The authors declare no conflict of interest.

## Supporting information



Supporting information

## Data Availability

The data that support the findings of this study are available from the corresponding author upon reasonable request.
