# Supplementary material for: Decoy Extracellular Vesicles Overcome Triple‐Negative Breast Cancer Heterogeneity via Membrane‐Cytoplasm‐Mitochondria Cascade Targeting
Source: Adv Sci (Weinh). 2025 Aug 4;12(40):e07975. doi: 10.1002/advs.202507975 (PMC12561465; doi:10.1002/advs.202507975)
Supplement: Supplementary file 1 — Supporting information [file ADVS-12-e07975-s001.docx]

**Supporting Information**

**Decoy Extracellular Vesicles Overcome Triple-Negative Breast Cancer Heterogeneity via Membrane-Cytoplasm-Mitochondria Cascade Targeting**

Chuanrong Chen^1†^*, Ming Shen^2^*^†^, Xiaofeng Wan^1^, Lili Sheng^1^, Na Hao^1^, Man Li^1^, Menglin Xu^1^, Yang He^1^*, Jiali Zhang^3^*,

^†^Chuanrong Chen and Ming Shen contributed equally to this work

^1^Department of Oncology, Yijishan Hospital of Wannan Medical College, Wuhu 240001, China.

^2^National Health Commission (NHC) Key Laboratory of Reproduction Regulation, Shanghai Institute for Biomedical and Pharmaceutical Technologies, Shanghai 200032, China.

^3^State Key Laboratory of Oncogenes and Related Genes, Shanghai Cancer Institute, Renji Hospital, School of Medicine, Shanghai Jiao Tong University, Shanghai 200032, China

^*^Corresponding authors: Jiali Zhang; E-mail: [jlzhang@shsci.org](mailto:jlzhang@shsci.org)

Chuanrong Chen; E-mail: crchen1227@163.com

Ming Shen; E-mail: shenming711@163.com

Yang He; E-mail: hy030122@163. com


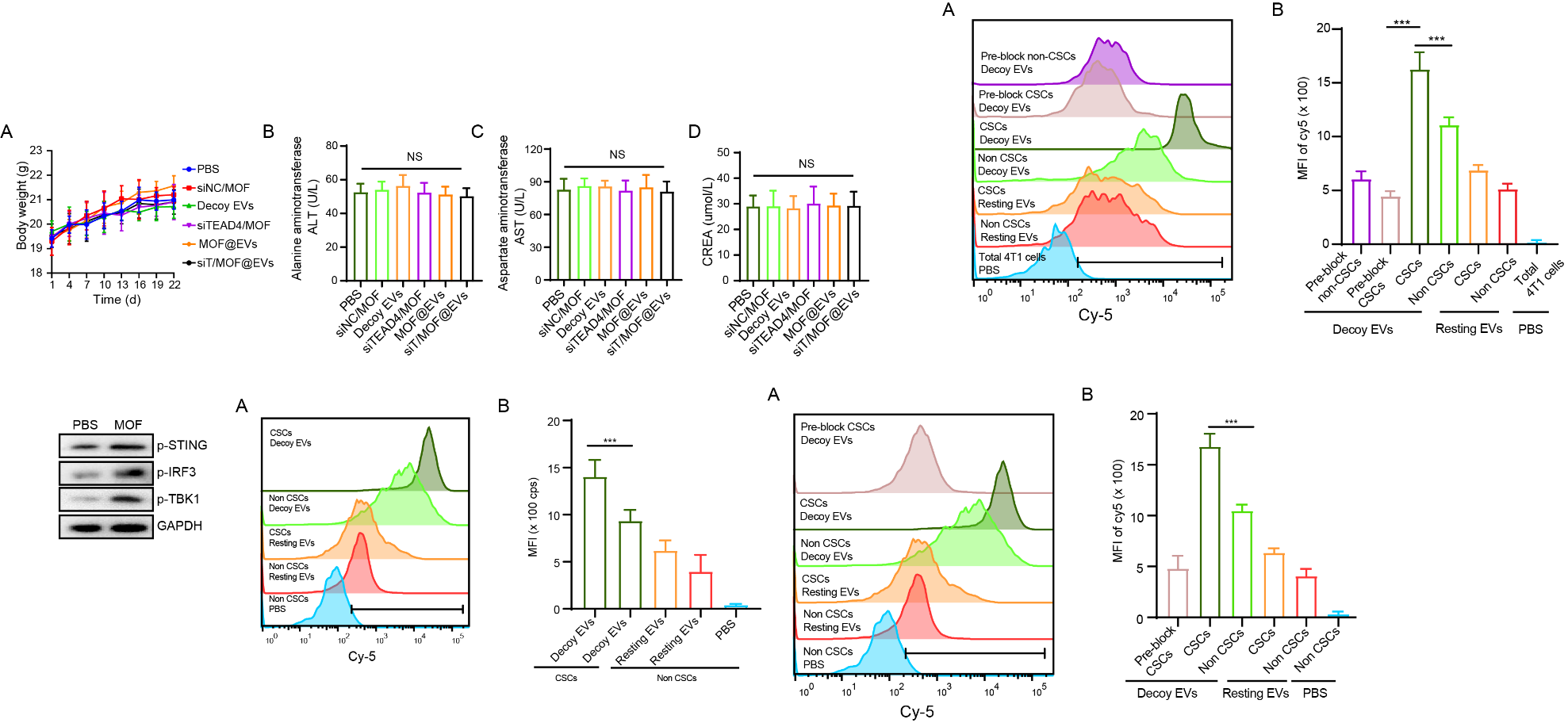


**Figure S1.** (A) Flow cytometry analysis of decoy EVs or resting EVs uptake by Pre-block CSCs, Pre-block non-CSCs (Pre-block EVs-surface PD-1, TIGIT, and Tim-3 with corresponding antibodies), CSCs or non-CSCs (non-cancer stem cells). (B) Quantitative analysis of mean fluorescence intensity (MFI) in A.


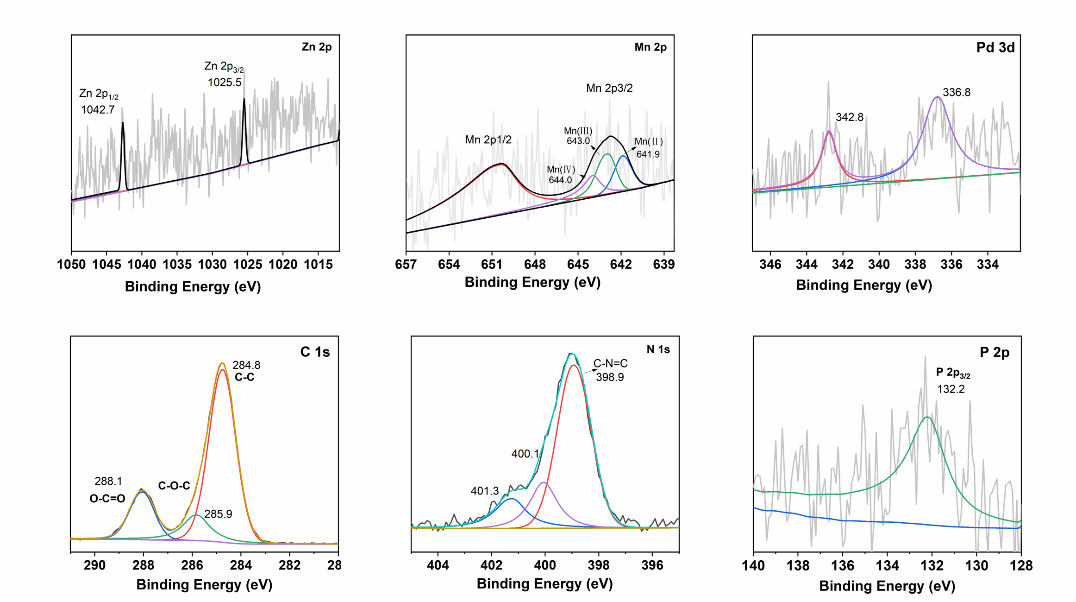


**Figure S2.** X-ray Photoelectron Spectroscopy (XPS) patterns of siRNA/MOF.


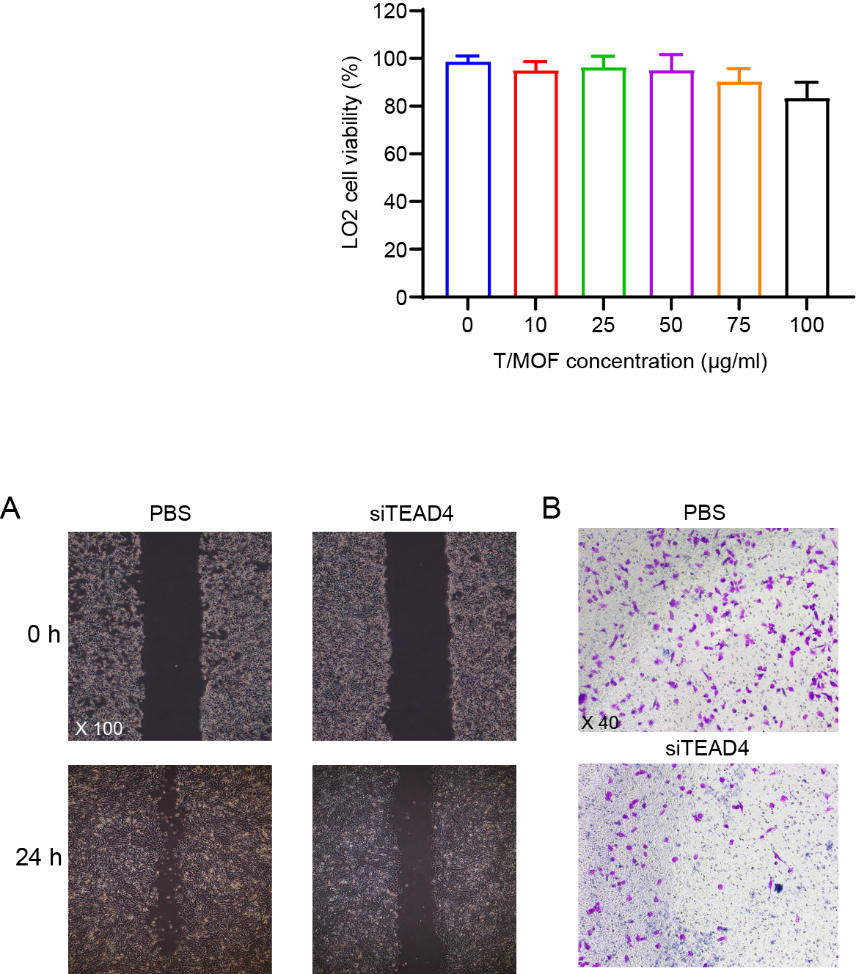


**Figure S3.** Human liver normal cell line LO2 were treated with various concentrations of T/MOF for 48 h. The viability of LO2 cells was measued by CCK-8.


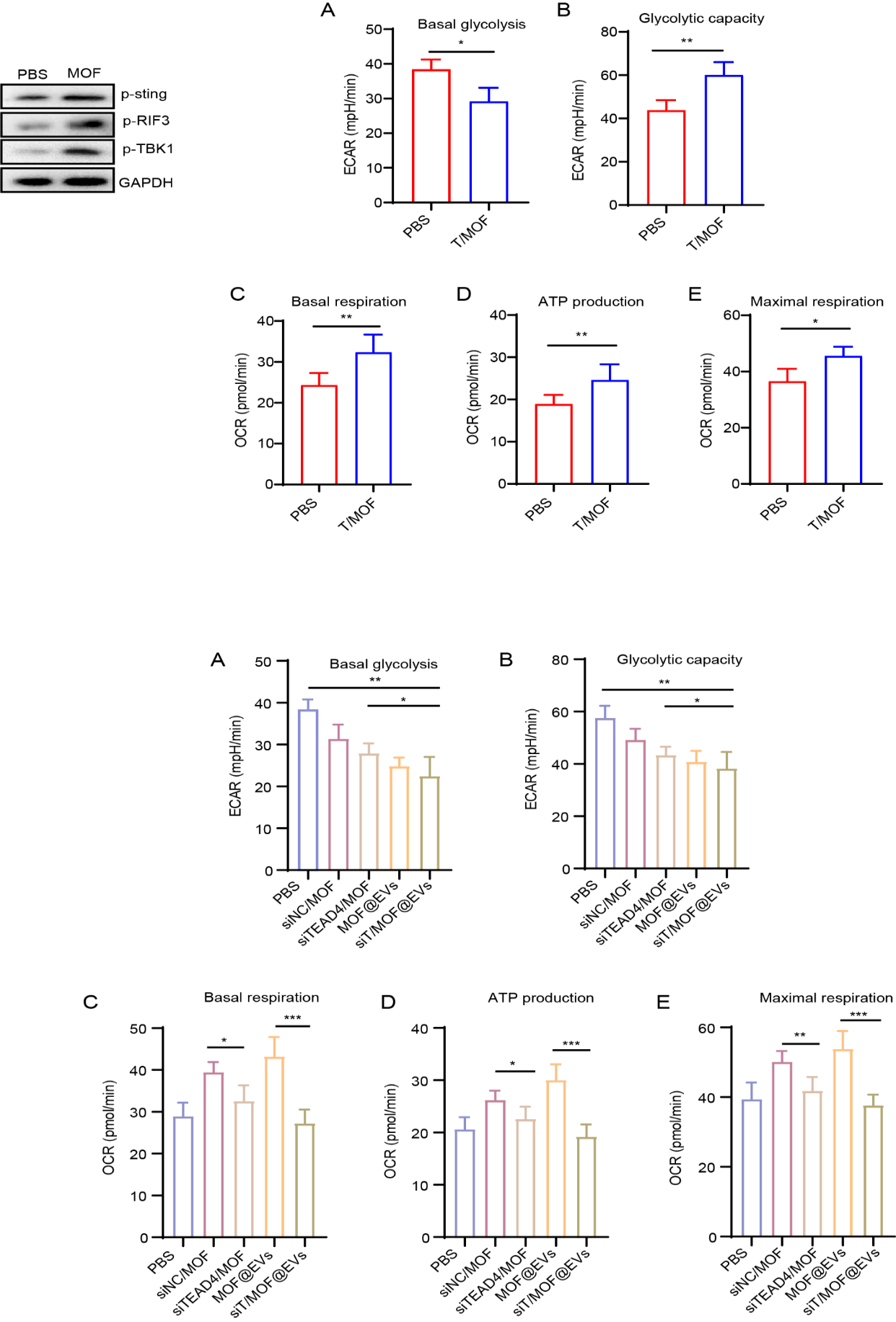


**Figure S4.**（A,B）Effects of T/MOF on basal glycolysis (A) and maximum glycolysis (B) levels in 4T1 cells.（C-E）Effects of T/MOF on basal respiration (C), ATP production (D) and Maximal respiration (E) in 4T1 cells.


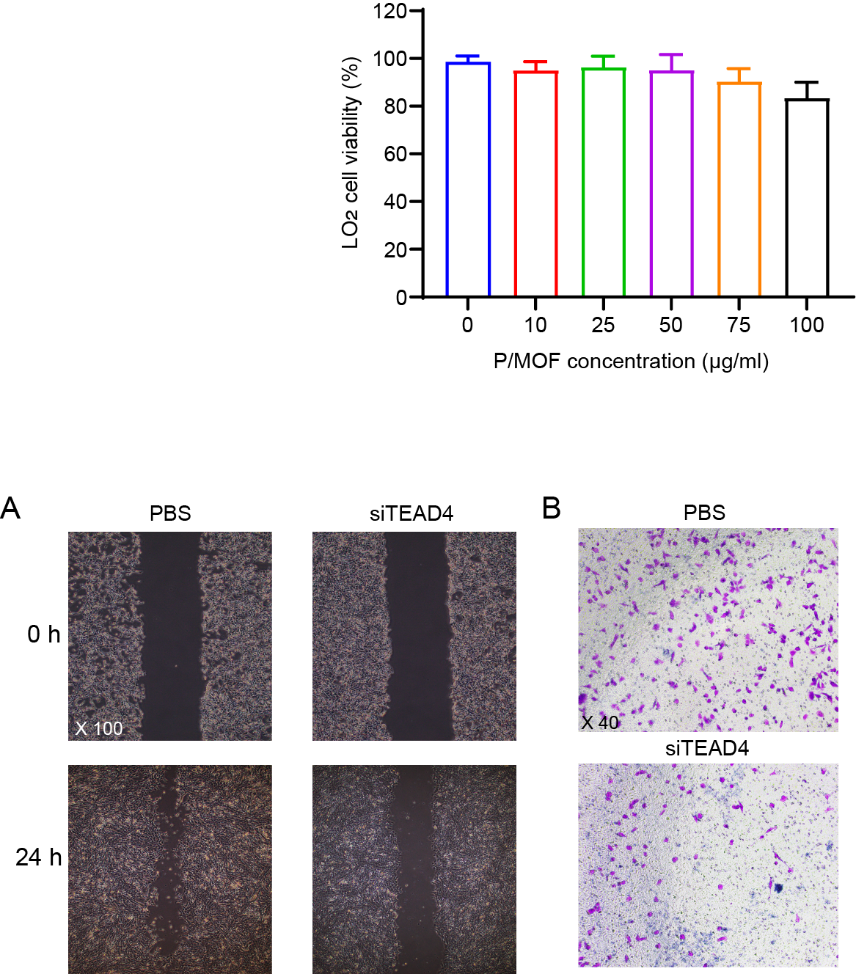


**Figure S5.** (A) Scratch assay to examine the migratory ability of 4T1 cells treated with siTEAD4 for 48h. (B) Transwell assay to evaluate the invasive of 4T1 cells treated with siTEAD4 for 48h.


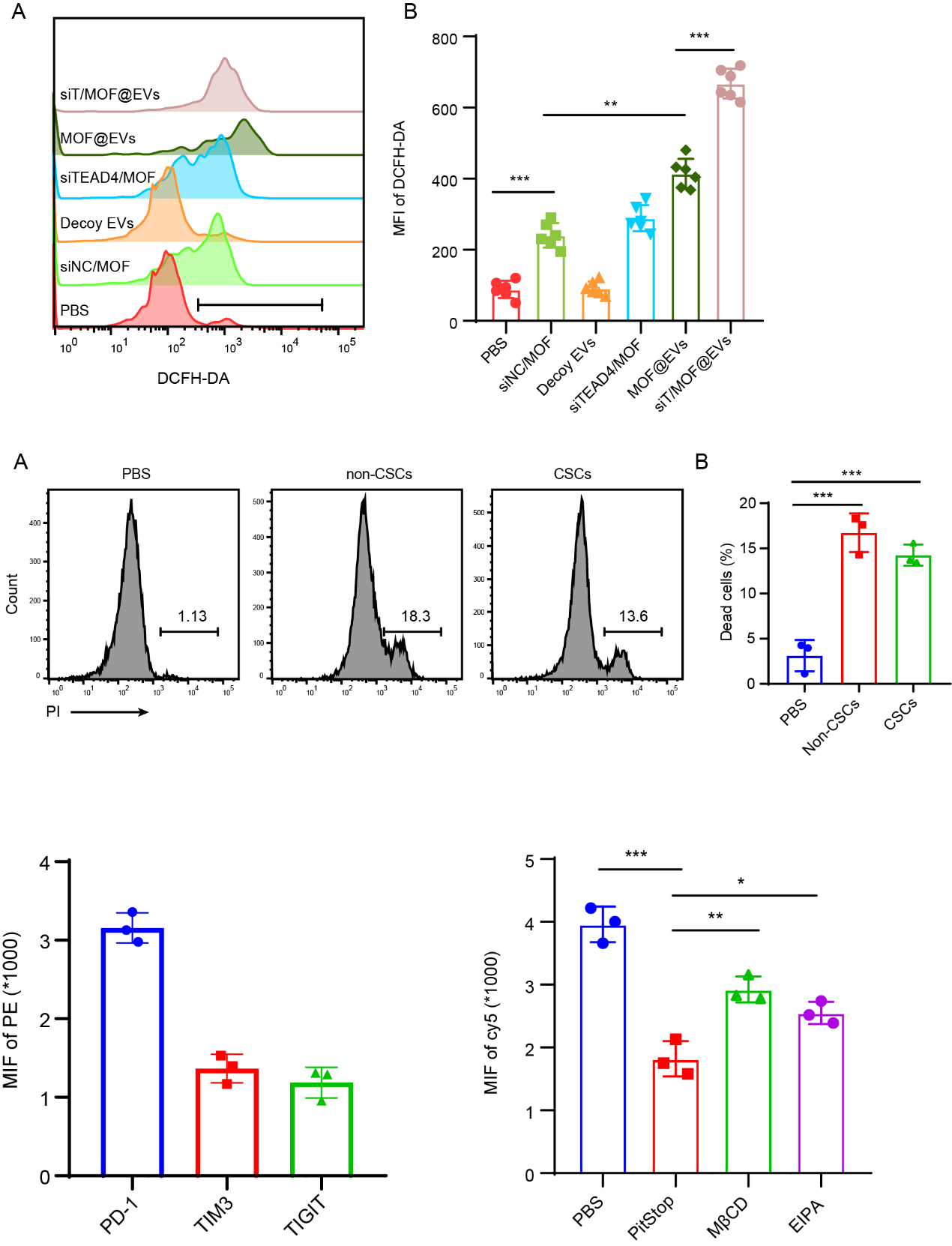


**Figure S6.** (A) Flow cytometry examines the cell death of 4T1 cells treated with siTEAD4-lipo2000 for 48h. (B) The statistics analysis of (A).


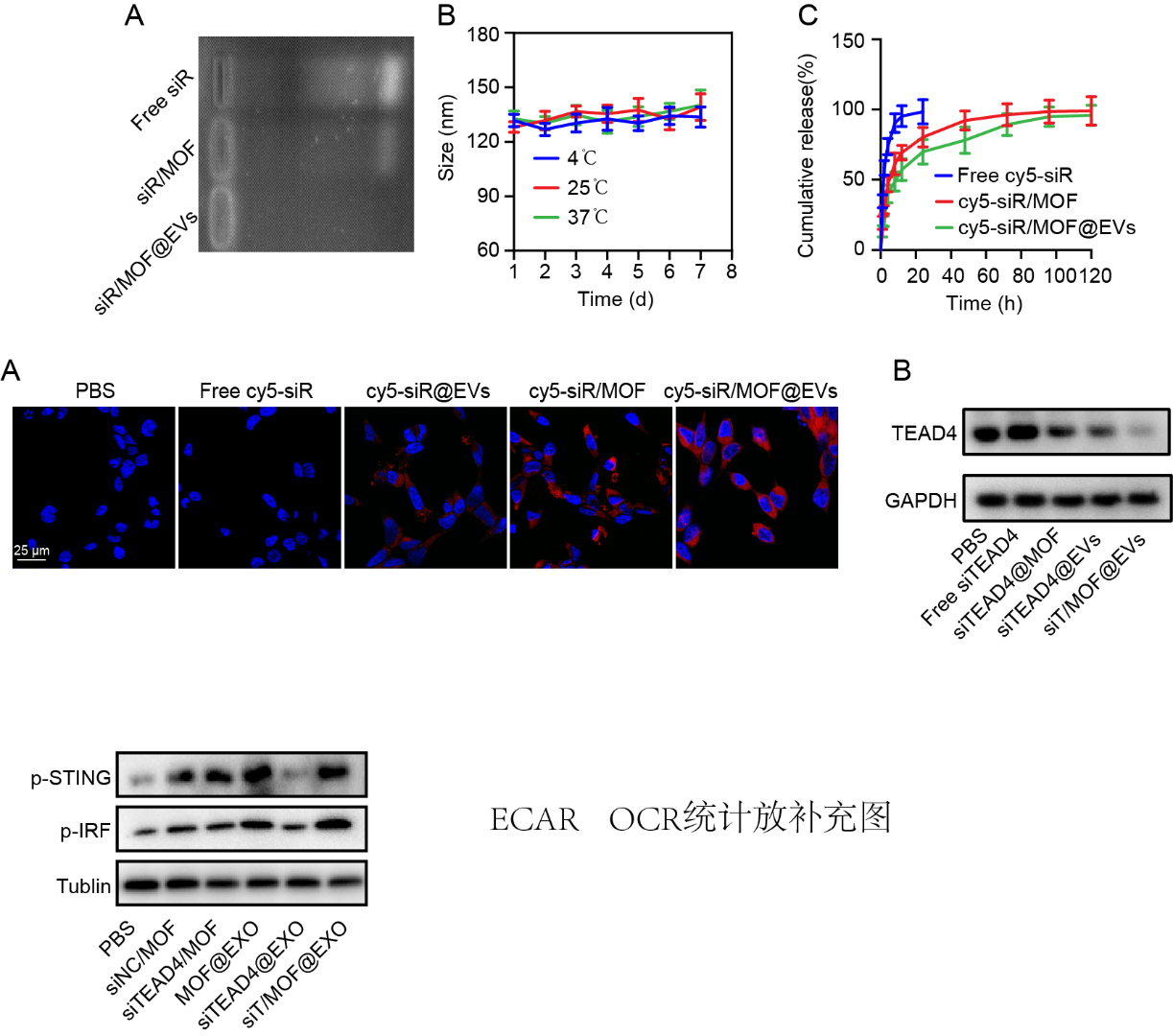


**Figure S7.** (A) Validation of siRNA loading efficiency of siR/MOF@EVs by agarose gel electrophoresis.（B）Particle size change of under different temperature conditions within a week.


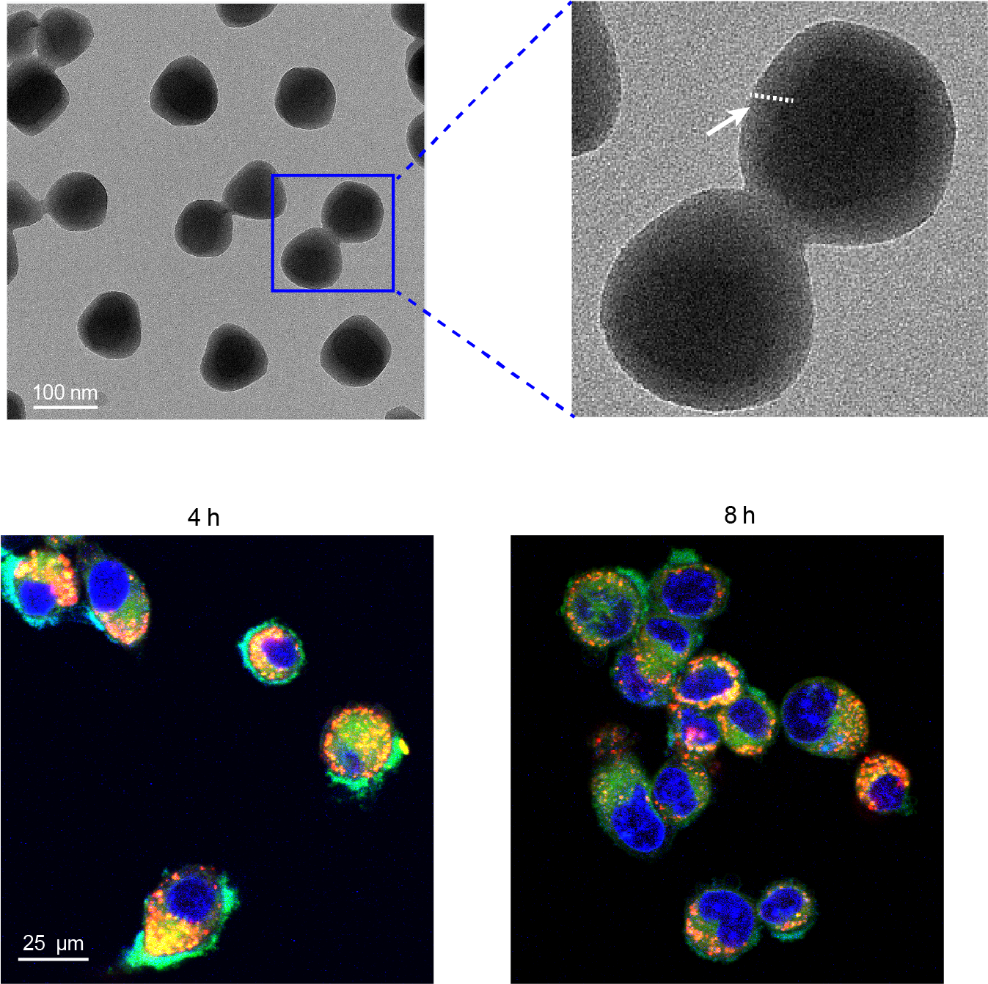


**Figure S8.** TEM analysis of siT/MOF@EVs.


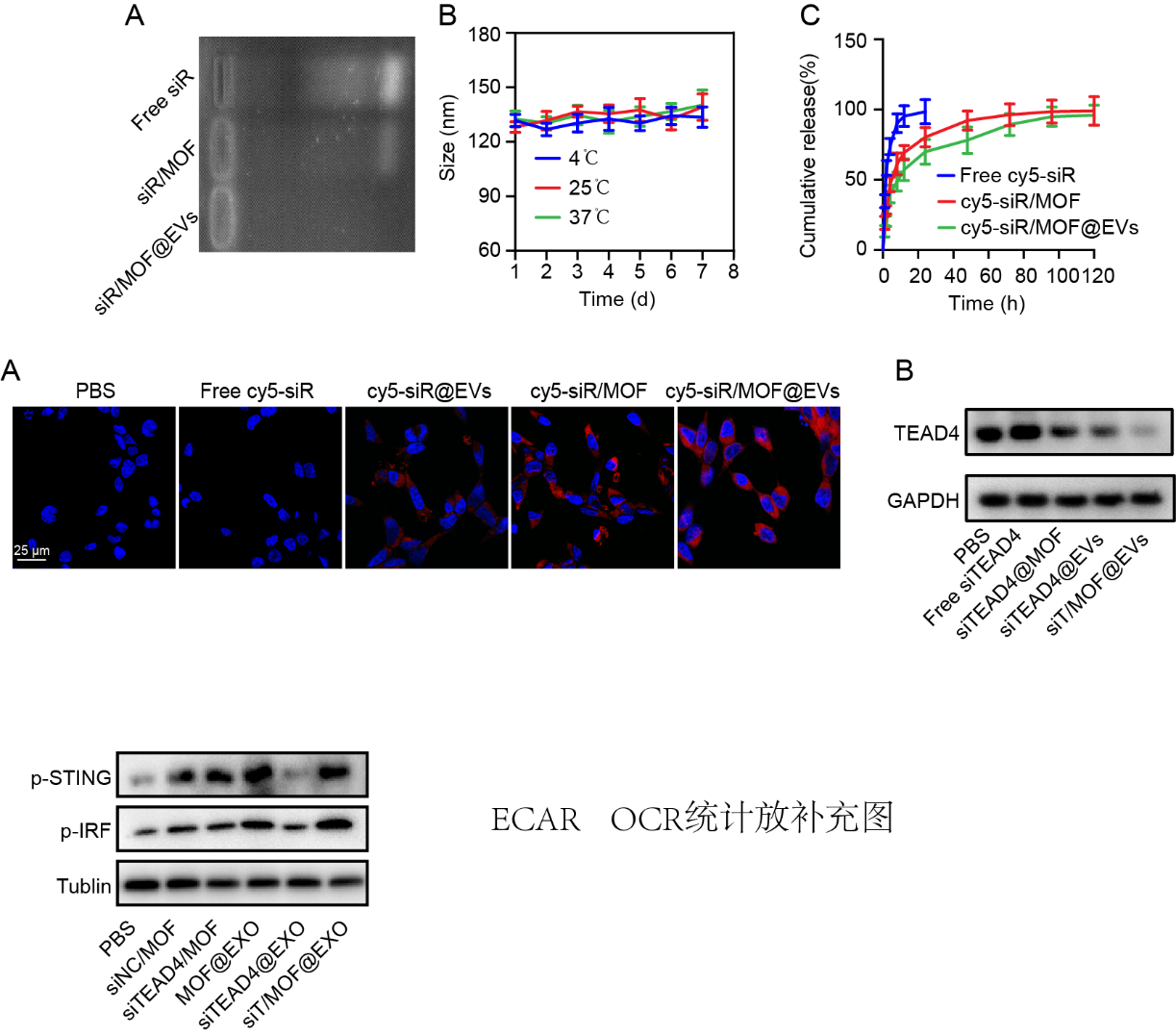


**Figure S9.** 4T1 cells were treated with PBS, free cy5-siR, cy5-siR@EVs, cy5-siR@MOF, cy5-siR/MOF@EVs for 6 h. (A) Cellular uptake was detected by CLSM. (B) Knockdown efficiency of TEDA4 protein in 4 T1 cells treated with siT/MOF@EVs for 48h.


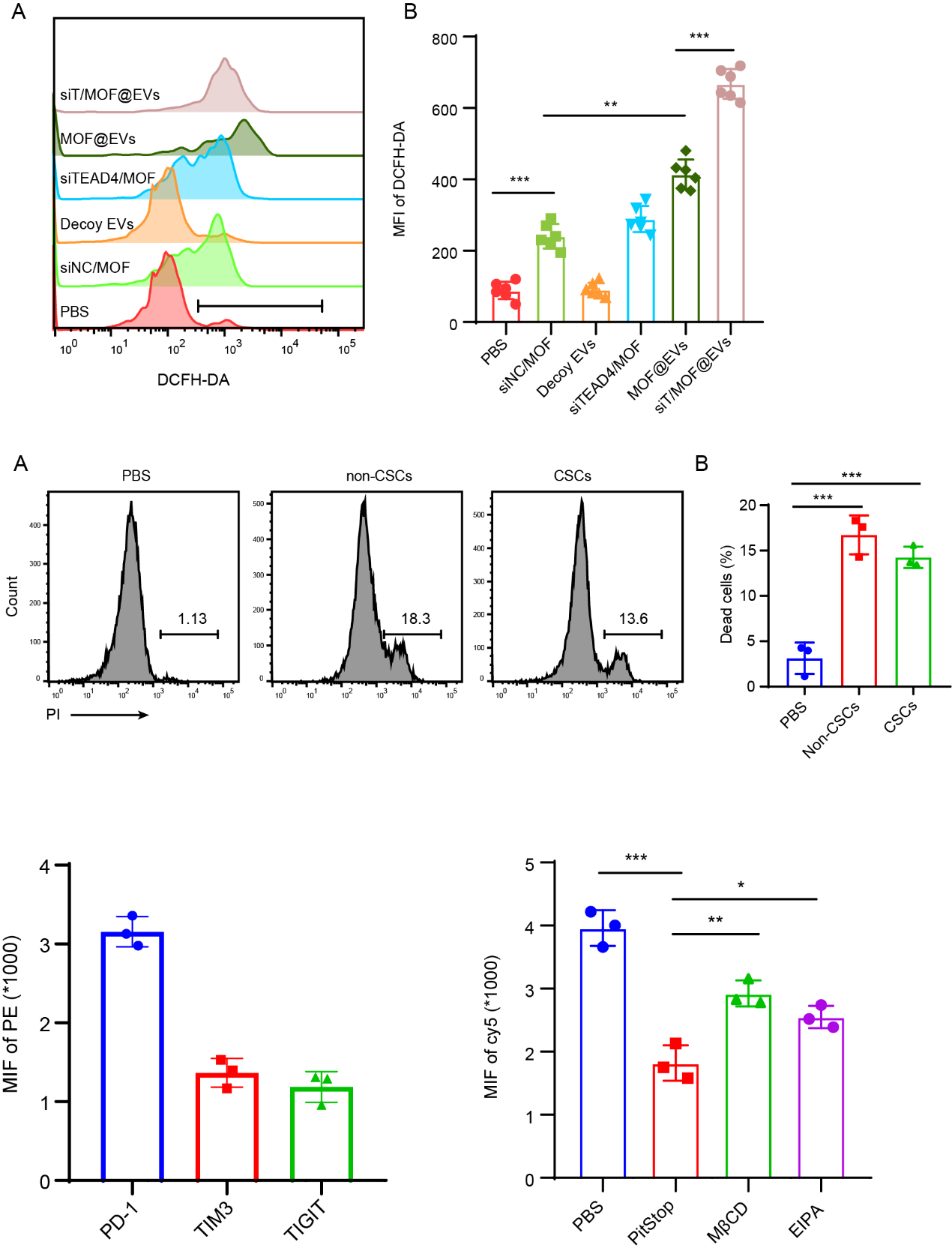


**Figure S10.** Quantitative analysis of NPs internalization upon different treatment.


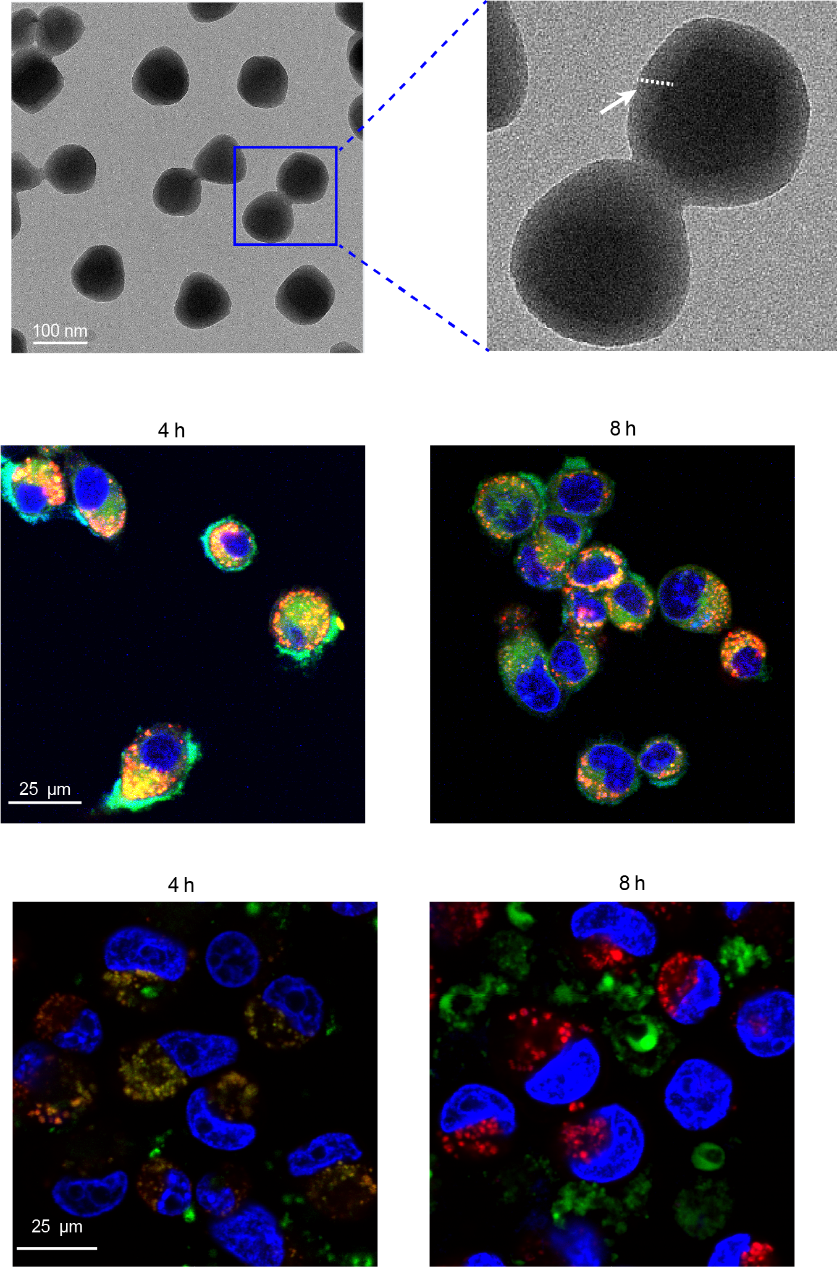


**Figure S11.** Fluorescent visualization of siRNA localization in 4T1 cells 4 and 8 hours after incubation with siT/MOF@EVs (scale bar, 25 μm; siRNA, green; nuclei, blue; endosomes, red).


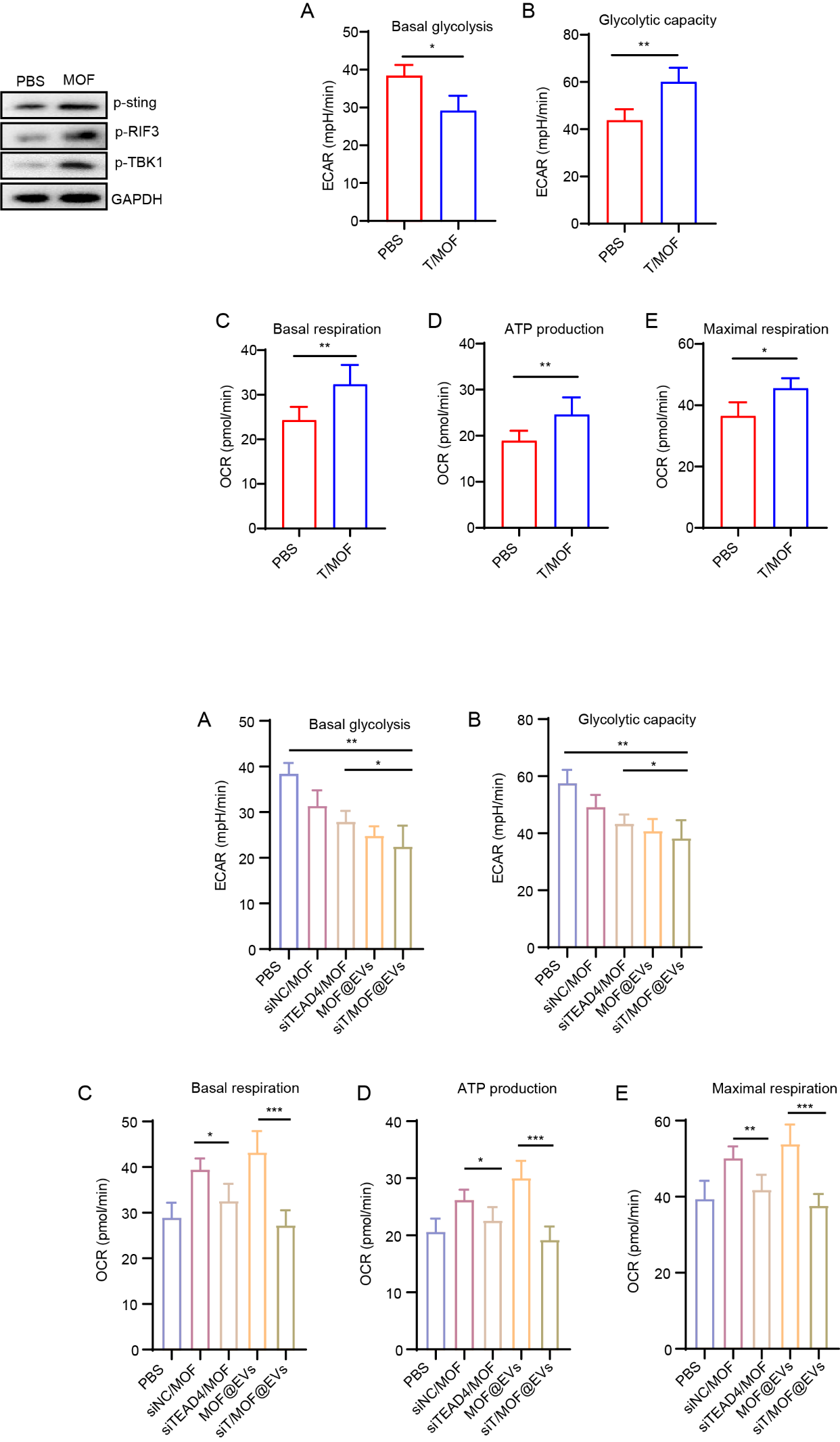


**Figure S12.**（A,B）Effects of siT/MOF@EVs on basal glycolysis (A) and maximum glycolysis (B) levels in 4T1 cells.（C-E）Effects of siT/MOF@EVs on basal respiration (C), ATP production (D) and Maximal respiration (E) in 4T1 cells.


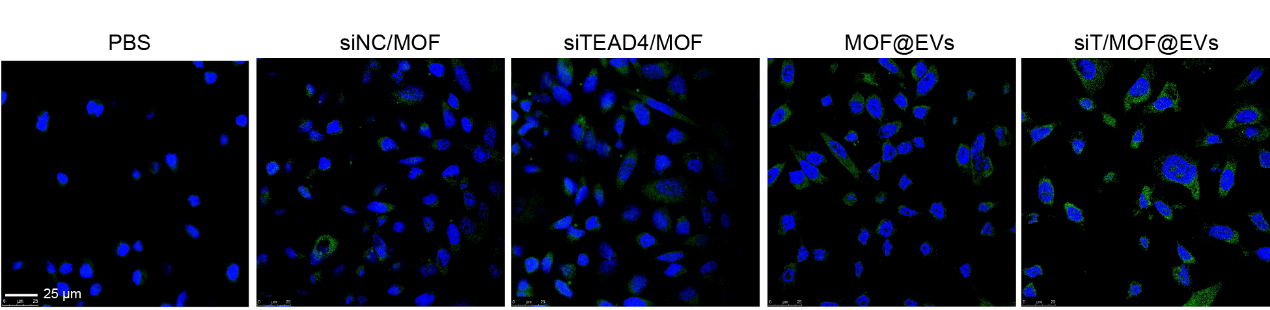


**Figure S13.** The CRT in 4T1 cell line was detected by immunofluorescence staining treated with indicated drugs for 48 h.


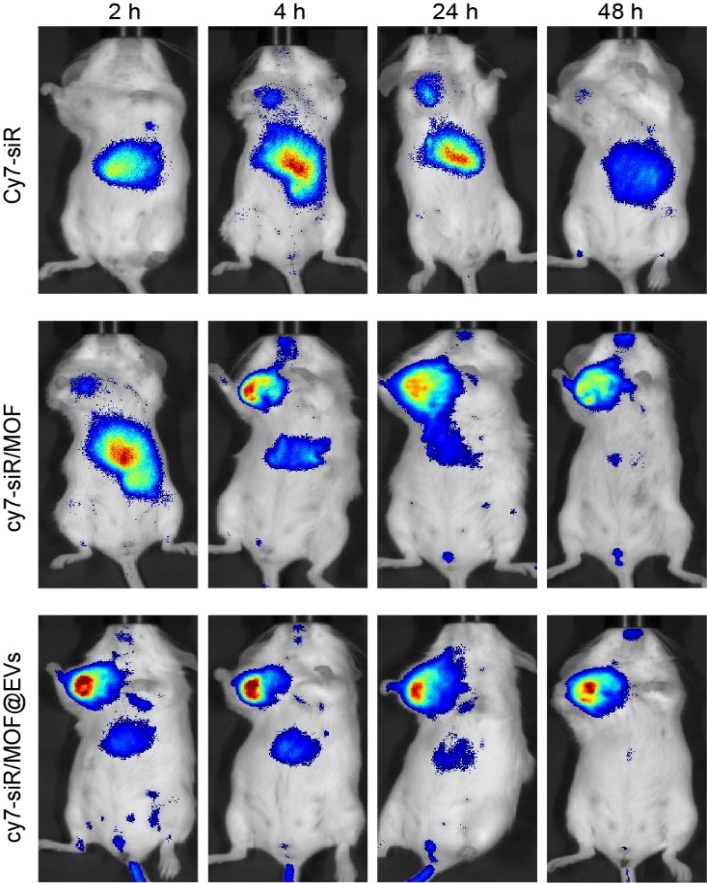


**Figure S14.** *In vivo* fluorescence image of orthostatic 4T1 tumor bearing mice at 2h, 8h, 24h and 48h after intravenous administration of free cy7-siR, cy7-siR/MOF or cy7-siR/MOF@EVs.


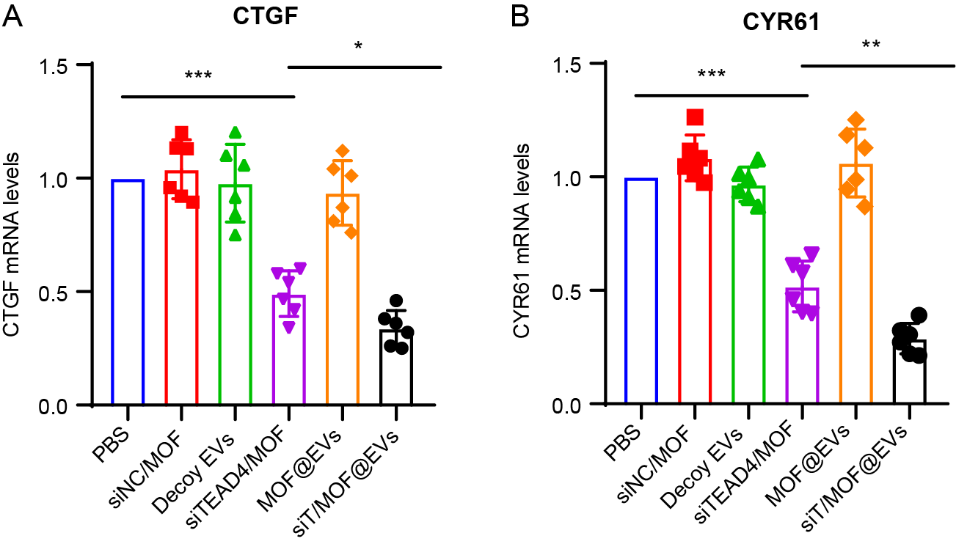


**Figure S15.** qPCR analysis of CTGF and CYR61 expression in tumor tissues.


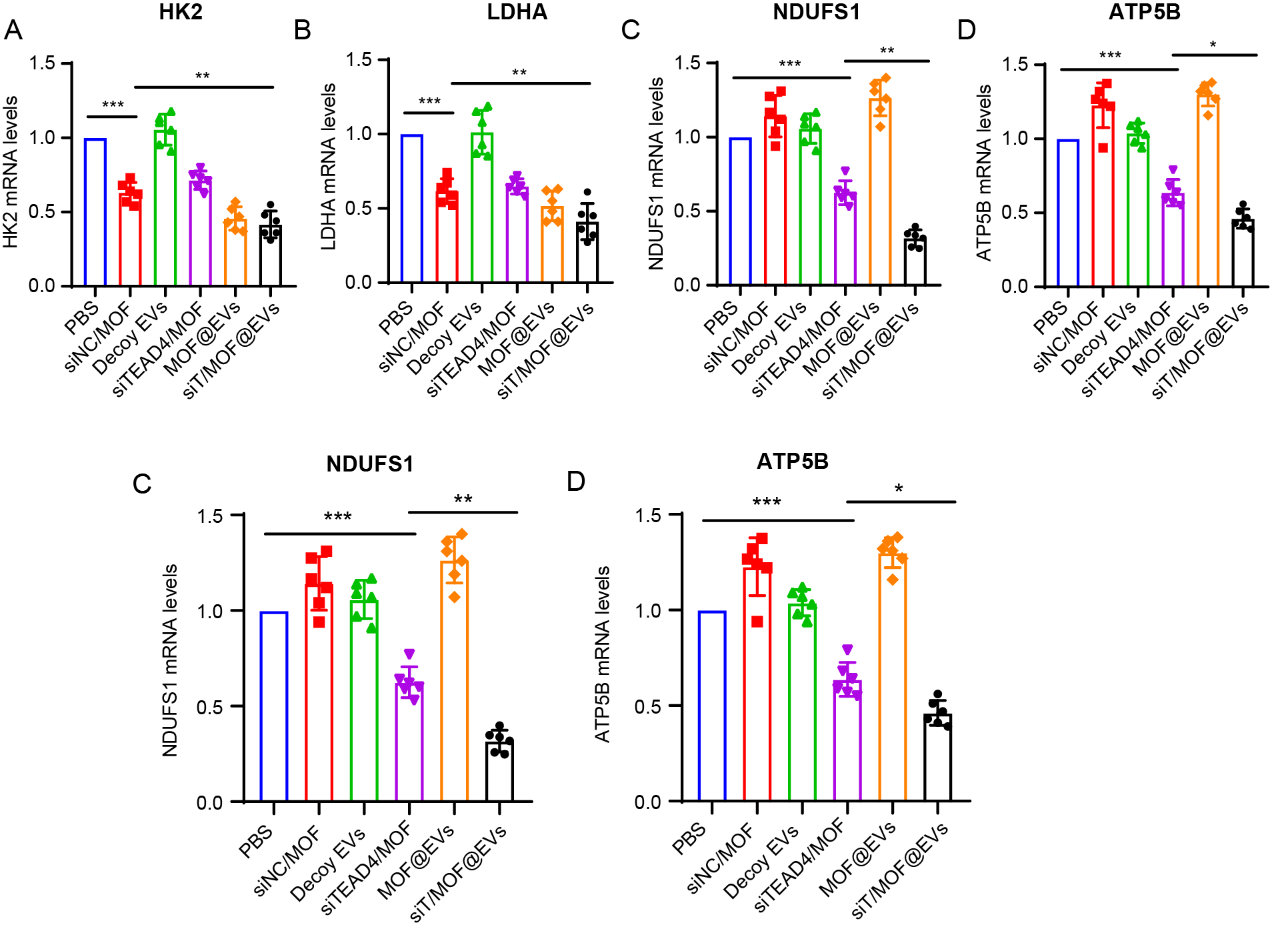


**Figure S16.** qPCR analysis of the key genes of glycolysis and oxidative phosphorylation expression in tumor tissues.

**
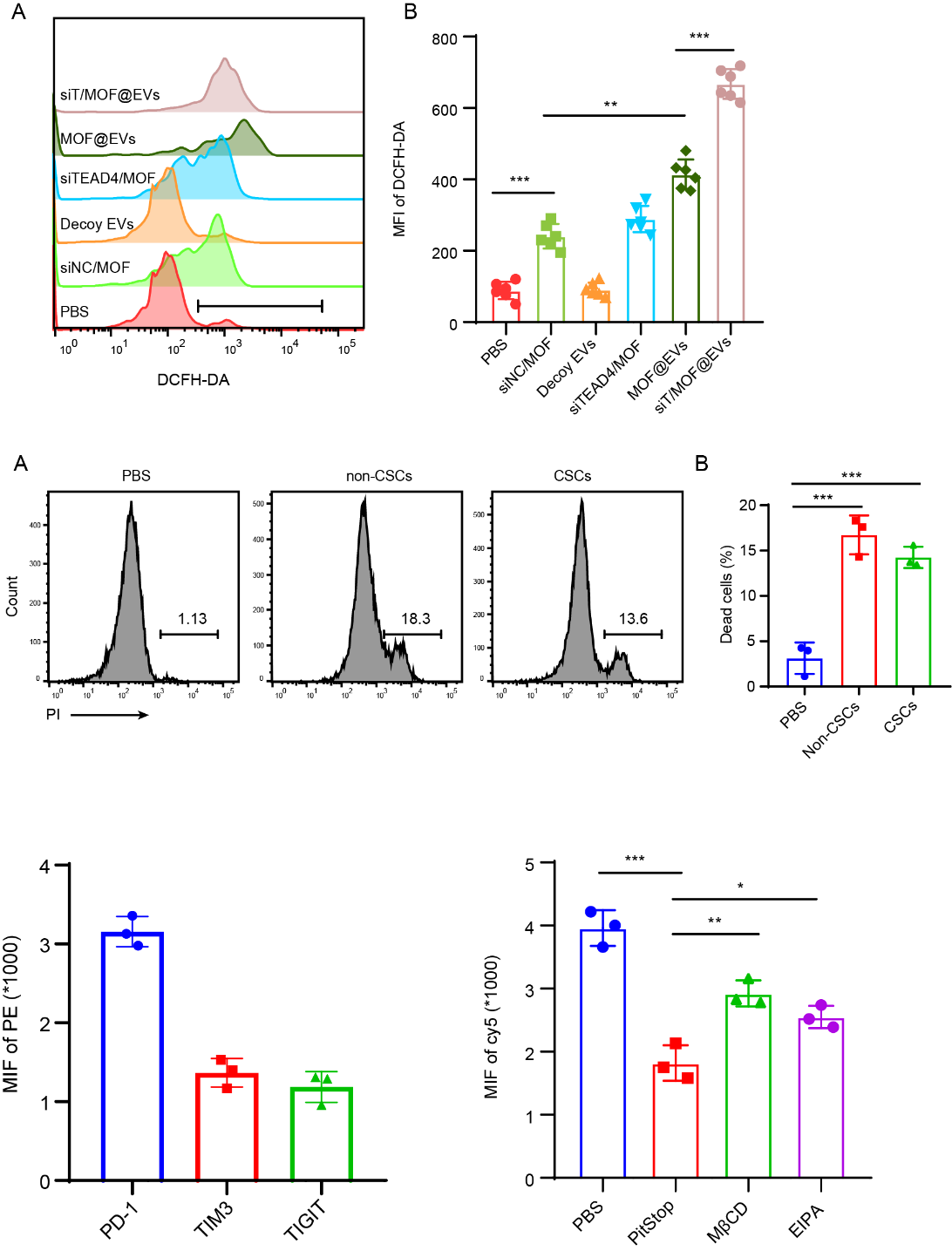
**

**Figure S17.** ROS level in single cells suspension of tumor tissues.


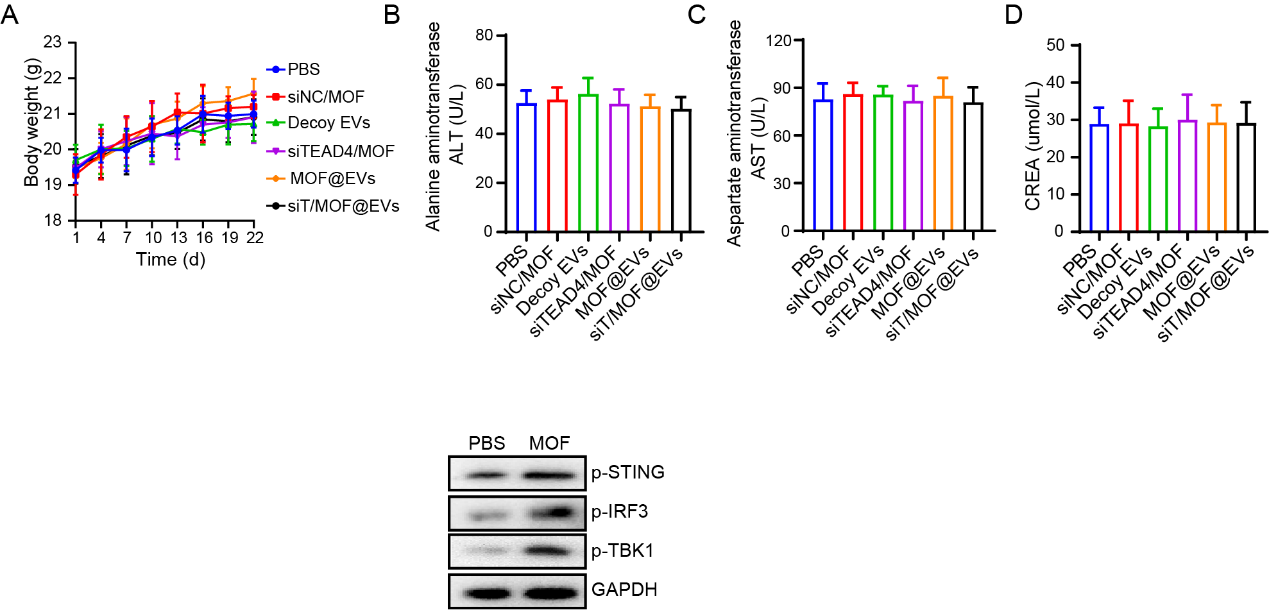


**Figure S18.** (A) Change curve of mice weight in each treatment group during therapy. (B-D) Serum ALT (B), AST (C) and CREA (D) levels of mice in each treatment group after treatment.


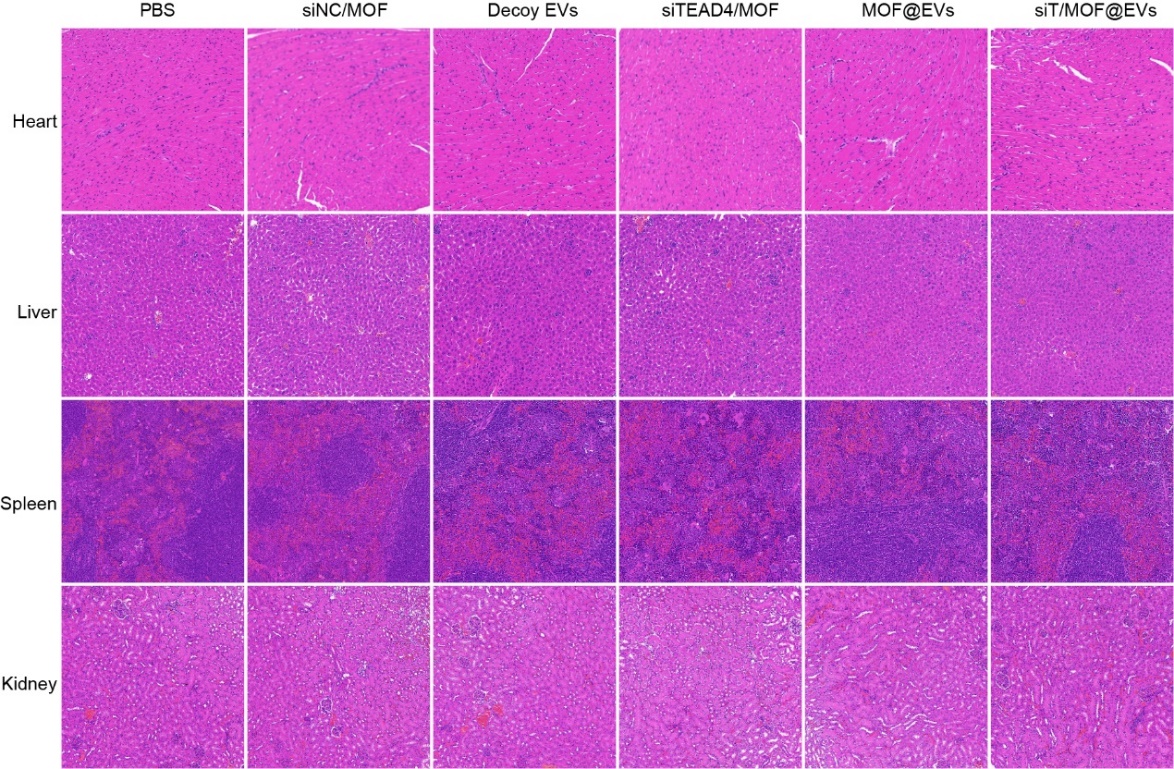


**Figure S19.** H&E images of major organs in mice following treatment with either PBS, siNC/MOF, decoy EVs, siTEAD4/MOF, MOF/EVs or siT/MOF@EVs.


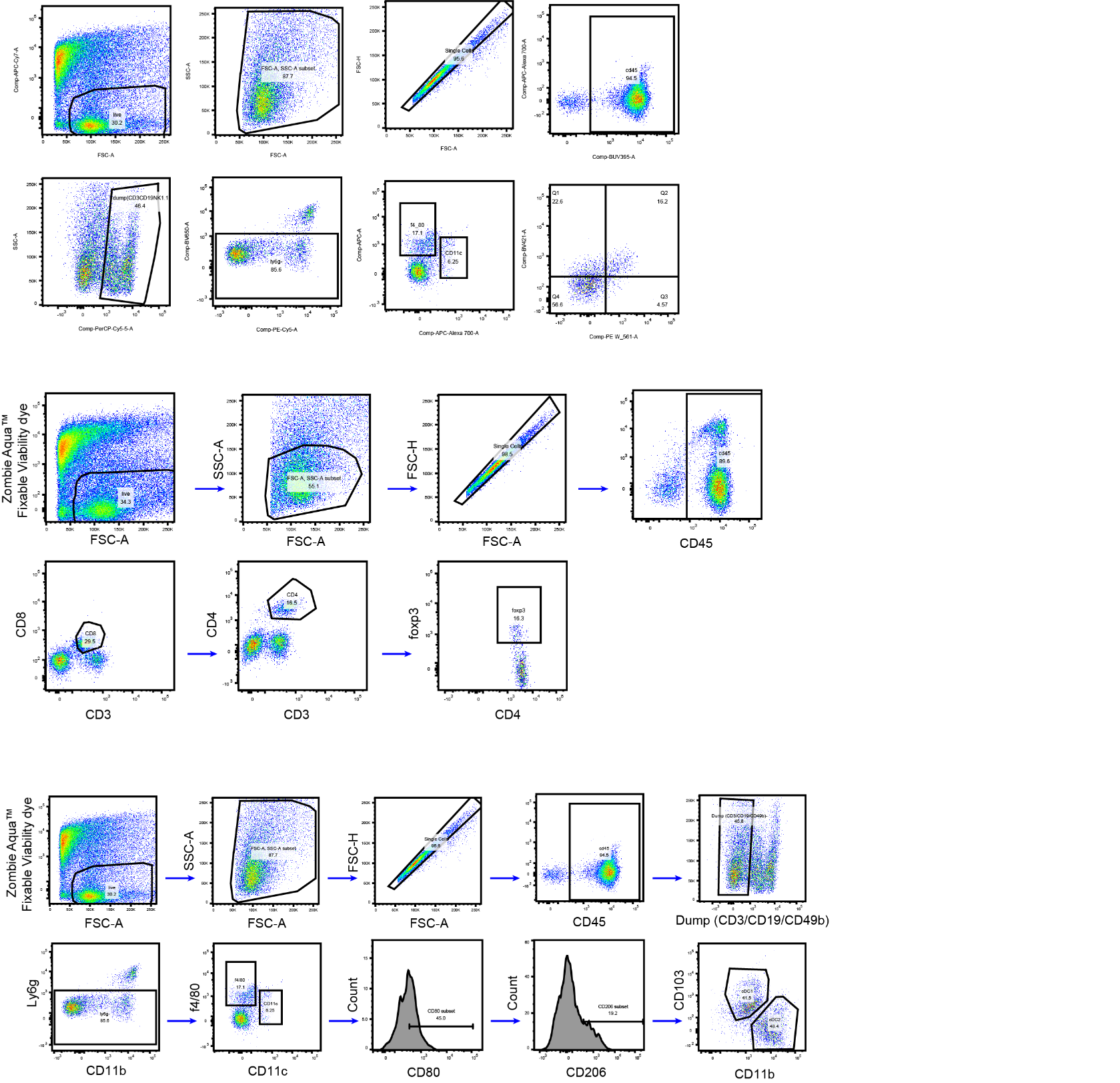


**Figure S20.** Flow cytometry analysis of cCD1 and cCD2 cells gate strategy.


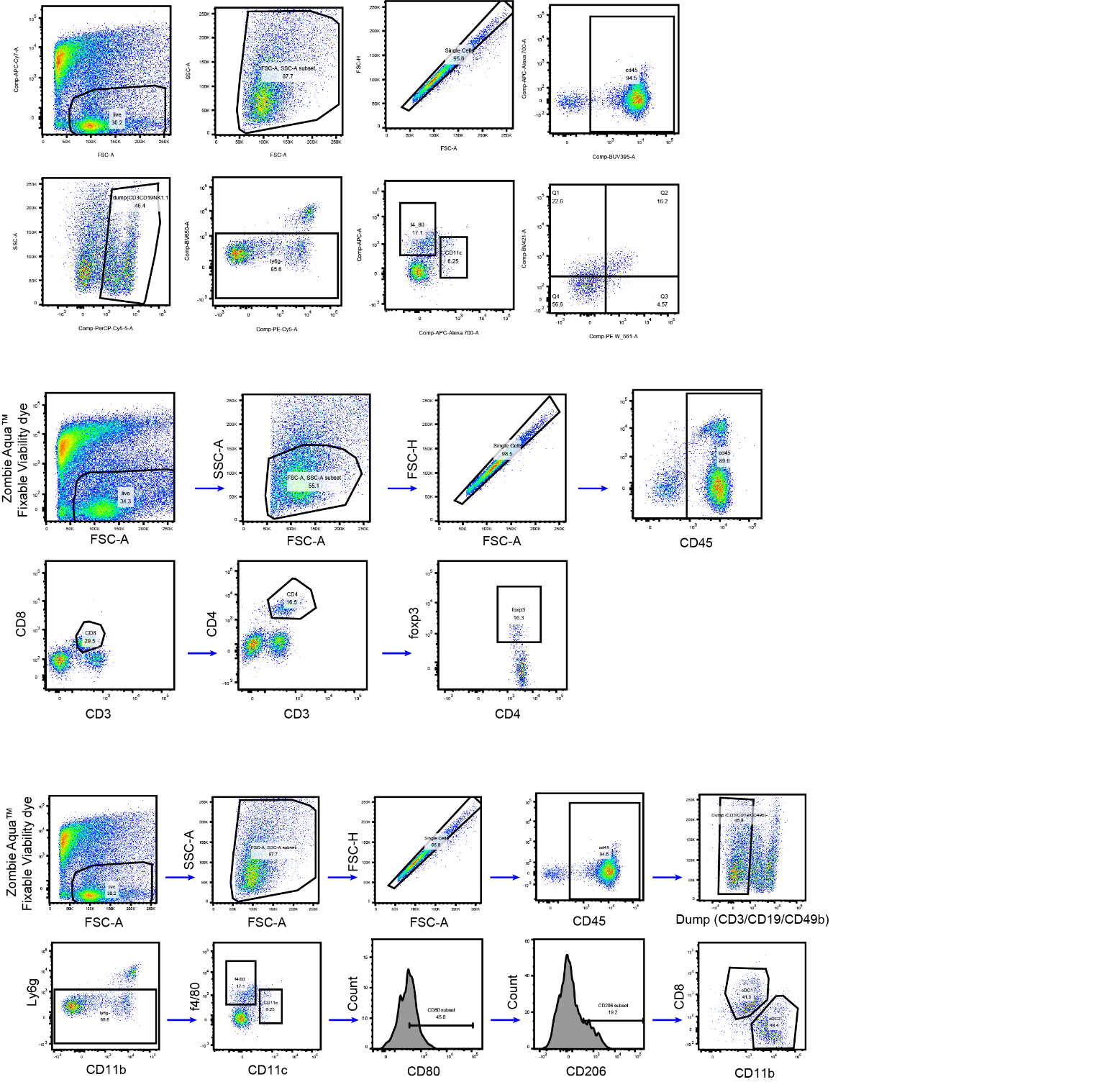


**Figure S21.** Flow cytometry analysis of CD8^+^ and CD4^+^ T cells gate strategy.


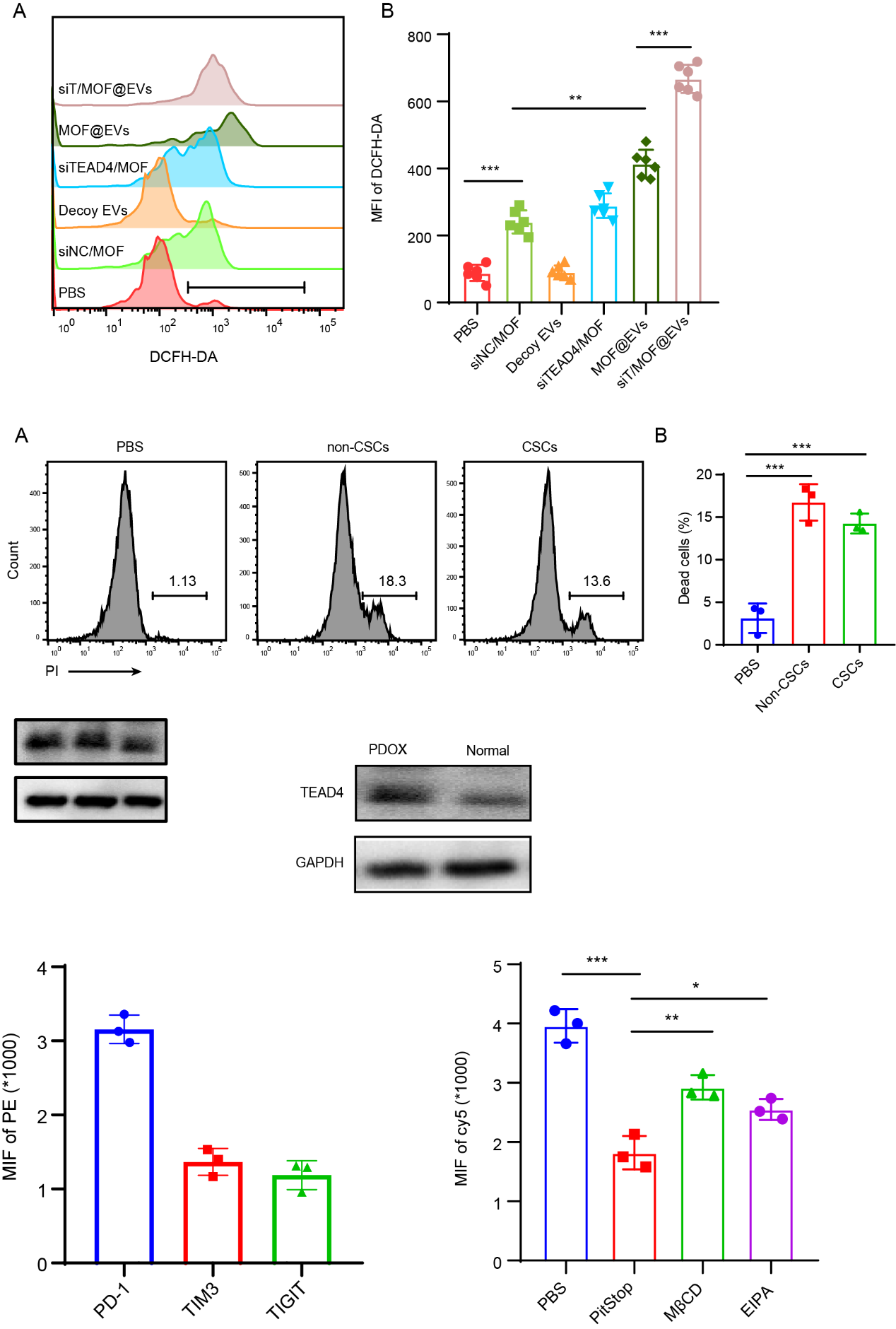


**Figure S22.** WB analysis of TEAD4 expression in organoids and adjacent cancer tissues.

**Table S1.** Source and clone number of antibodies

| Antibodies | Clone | Source |
| --- | --- | --- |
| Anti-mouse CD274 (PD-L1) antibody | 10F.9G2 | Biolegend |
| Anti- mouse PD-1 antibody | 29F.1A12 | Biolegend |
| Anti-mouse CD3 antibody | 17A2 | Biolegend |
| Anti-mouse CD28 antibody | 37.51 | BioLegend |
| Anti-mouse CD8a antibody | 53-6.7 | BioLegend |
| Anti-mouse CD25 antibody | 3C7 | BioLegend |
| Anti-mouse CD4 antibody | GK1.5 | BioLegend |
| Anti-mouse/human CD44 antibody | IM7 | BioLegend |
| Anti-mouse Tim3 antibody | B8.2C12 | Biolegend |
| Anti-mouse CD62L antibody | MEL-14 | Biolegend |
| Anti-human/mouse Ki-67 antibody | 11F6 | Biolegend |
| Anti-human/mouse Granzyme B antibody | GB11 | Biolegend |
| Anti-mouse CD11c antibody | N418 | BioLegend |
| Anti-mouse CD80 antibody | 16-10A1 | Biolegend |
| Anti-mouse CD86 antibody | A17199A | BioLegend |
| Anti-mouse CD45 antibody | 30-F11 | BioLegend |
| Anti-mouse TIGIT antibody | A117200C | BioLegend |
| Anti-mouse/human CD11b antibody | M1/70 | BioLegend |
| Anti-mouse CD19 antibody | 1D3/CD19 | BioLegend |
| Anti-mouse CD49 antibody | HMα1 | BioLegend |
| Anti-mouse Ly6g antibody | 1A8 | BioLegend |
| Anti-mouse CD206 antibody | C068C2 | BioLegend |
| Anti-mouse Foxp3 antibody | R16-715 | BD Pharmingen™ |
| Anti-mouse/human TEAD4 antibody | Q15561 | Affinity |
| Anti-mouse/human Tubulin antibody | P07437 | Affinity |
| Anti- mouse PD-1 antibody | EPR20665 | Abcam |
| Anti-mouse/human HK2 antibody | EPR200839 | Abcam |
| Anti- mouse Alix antibody | EPR23653-32 | Abcam |
| Anti- mouse/human Tsg101 antibody | E6V1X | Cell Signaling Technology |
| Anti-mouse/human GAPDH antibody | 14C10 | Cell Signaling Technology |
| Anti-mouse/human Calreticulin antibody | D3E6 | Cell Signaling Technology |
